# The Expenditures for Academic Inpatient Care of Inflammatory Bowel Disease Patients Are Almost Double Compared with Average Academic Gastroenterology and Hepatology Cases and Not Fully Recovered by Diagnosis-Related Group (DRG) Proceeds

**DOI:** 10.1371/journal.pone.0147364

**Published:** 2016-01-19

**Authors:** Daniel C. Baumgart, Marie le Claire

**Affiliations:** 1 Inflammatory Bowel Disease Center, Department of Gastroenterology and Hepatology, Charité Medical School, Humboldt-University of Berlin, Berlin, Germany; 2 Enterprise Controlling, Charité Medical School, Humboldt-University of Berlin, Berlin, Germany; Harvard Medical School, UNITED STATES

## Abstract

**Background:**

Crohn’s disease (CD) and ulcerative colitis (UC) challenge economies worldwide. Detailed health economic data of DRG based academic inpatient care for inflammatory bowel disease (IBD) patients in Europe is unavailable.

**Methods:**

IBD was identified through ICD-10 K50 and K51 code groups. We took an actual costing approach, compared expenditures to G-DRG and non-DRG proceeds and performed detailed cost center and type accounting to identify coverage determinants.

**Results:**

Of all 3093 hospitalized cases at our department, 164 were CD and 157 UC inpatients in 2012. On average, they were 44.1 (CD 44.9 UC 43.3 all 58) years old, stayed 10.1 (CD 11.8 UC 8.4 vs. all 8) days, carried 5.8 (CD 6.4 UC 5.2 vs. all 6.8) secondary diagnoses, received 7.4 (CD 7.7 UC 7 vs. all 6.2) procedures, had a higher cost weight (CD 2.8 UC 2.4 vs. all 1.6) and required more intense nursing. Their care was more costly (means: total cost IBD 8477€ CD 9051€ UC 7903€ vs. all 5078€). However, expenditures were not fully recovered by DRG proceeds (means: IBD 7413€, CD 8441€, UC 6384€ vs all 4758€). We discovered substantial disease specific mismatches in cost centers and types and identified the medical ward personnel and materials budgets to be most imbalanced. Non-DRG proceeds were almost double (IBD 16.1% vs. all 8.2%), but did not balance deficits at total coverage analysis, that found medications (antimicrobials, biologics and blood products), medical materials (mostly endoscopy items) to contribute most to the deficit.

**Conclusions:**

DRGs challenge sophisticated IBD care.

## Introduction

Due to their increasing incidence and early age peaks in the 2^nd^ and 3^rd^ decade and commonly associated extraintestinal manifestations the systemic conditions Crohn’s disease[1] (CD) and ulcerative colitis[2] (UC) substantially impact on patient’s personal and professional lives and challenge economies and healthcare systems worldwide. The complete economic burden has been estimated at 8.1 to 14.9 for UC and 10.9 to 15.5 billion US$ for CD, respectively in the United States and 12.5 to 29.1 for UC and 2.1 to 16.7 billion € for CD annually in Europe[3, 4].

Several countries in Europe[5–7], North America[8, 9] and Australia[10] have decided to introduce diagnosis related group (DRG) billing systems hoping to achieve better cost control. The origins of the DRG system date back to a collaborative project at the Yale University School of Management and School of Public Health[11]. European DRG systems have implemented different design options, are generally more detailed than the Medicare DRG system aiming to better distinguish among patients with less and more complex conditions and include physician salaries and re-admission[12]. DRG was introduced as a MeSH term in PubMed in 1991.

DRG introduction, an overall very low investment ratio in hospitals and passing of European Union legislation[13] that dictates a strict separation of business (i.e. hospital) from non-business (i.e. academic) activities put most academic medical centers into financial turmoil[6, 14]. This resulted in extreme scrutiny towards medical specialties caring for cost intensive patients.

It is currently unknown, if and how actual expenses are distributed and balanced with DRG proceeds in inflammatory bowel disease (IBD) inpatients at academic medical centers.

## Methods

The aim of our study was to compare actual cost and revenue with respective DRG proceeds for IBD cases and relate them to all other gastroenterology and hepatology inpatient cases within the same period. Moreover, we wanted to identify and analyze factors contributing to either excess (DRG proceeds higher than actual costs) or deficit (DRG proceeds lower than actual costs) coverage in IBD cases.

### Data source and identification of cases

All data were extracted from our medical center’s data warehouse running SAP NetWeaver Business Intelligence software[15] and other IT systems. In full conformity with all applicable federal privacy rules and regulations, no personally identifiable information was collected. From all inpatient service cases seen in 2012 at our department we identified inflammatory bowel disease patients using the German modification (ICD-10-GM)[16] of the WHO ICD-10[17] code groups for Crohn’s disease (K50) and ulcerative colitis (K51). Coding at out medical center is primarily done by specialized nurses and confirmed by physicians. No additional verification was performed for this research study. Data sets included age, gender, ICD-10-GM[16] coded main and secondary diagnoses, length of stay, procedures coded by the German modification (OPS)[18] of the International Classification of Health Interventions (ICHI)[19] and G-DRG, patient clinical complexity level (PCCL) [20]), cost weight (CW)[21], length of stay and discharge type.

### Calculation basis and accounting

All calculations were based on national coding rules[22] of the German Institute for the Hospital Remuneration System (InEK). The allocation of costs to a case followed a full-cost approach based on actual cost. Included were all cases, benefits and costs of the hospital under the remuneration framework of the G-DRG[23] system. The 2012 grouper (software to calculate diagnosis related groups–DRGs) and a base rate of € 2,985 were applied.

#### Cost type accounting

Prior to cost type accounting data were adjusted to exclude non-DRG relevant positions (e.g. other periods, extraordinary expenses or investments). Furthermore, all accounts were delineated for services compensated outside of the DRG system and those serving primary scientific research and teaching of medical students to meet EU academic accounting rules that prohibit subsidizing medical care through academic activities[13].

*Cost center accounting*—Cost centers were initially divided in direct (immediate patient care) and indirect (e.g. administration, facility management) cost centers. The settlement was based on the calculation set out in national coding guidelines[22] employing the equation method.

#### Cost unit accounting

Finally, the allocation of the costs of direct cost centers was carried out on an individual case basis and in the K50 and K51 case groups. Allocations were based on cost type- and center benchmarks. Direct costs were added to the cases based on information from the hospital information system (HIS) on used material costs, medications, medical imaging and operating room expenses. All other costs not fitting these categories (overheads) were distributed and added according to performance keys (e.g. operating room minutes, length of stay).

## Results

### Patient population

A total of 3093 individuals received full spectrum academic gastroenterology and hepatology inpatient care at our department in 2012. We identified 321 cases with inflammatory bowel disease. **Table 1** summarizes their demographics and key economic Figures. At a glance, IBD patients were on average fifteen years younger, stayed two (UC) to three days (CD) longer on their admissions, were sicker (CD > UC), required more intense nursing (CD > UC), received one to two more procedures (CD > UC) and generated almost twice the total cost compared with the average inpatient. The care of CD patients was more costly compared with UC and the average of all inpatients, but overall resulted in an excess coverage when DRG and non-DRG proceeds were included.

**Table 1:** Summary of key demographic and health economic Figures of inflammatory bowel disease cases versus all gastroenterology and hepatology academic inpatient service cases in 2012 calculated with a base rate of € 2985.

	Crohn‘s	Disease	Ulcerative	Colitis	IBD		All	
	Mean	∑	Mean	∑	Mean	∑	Mean	∑
**N**		164		157		321		3,093
**Age [years]**	44.9		43.3		44.1		58	
**Length of stay [days]**	11.8	1,928	8.4	1,321	10.1	1,625	8	24,615
**Cost Weight (CW)**	2.828	464	2.139	336	2.4835	400	1.594	4930
**PPR (Nursing Effort)**	1,516	256,058	909	142,758	1,213	199,408	1,029	3,182,588
**No. of Secondary Diagnoses**	6.4		5.2		5.8		6.8	
**No. of Procedures**	7.7		7		7.35		6.2	
**Total Costs [€]**	9,051	1,484,367	7,903	1,240,767	8,477	1,362,567	5,078	15,704,871
**Daily Costs [€]**	770	126,264	946	148,476	858	13,370	638	1,973,397
**DRG Proceeds [€]**	8,441	1,384,389	6,384	1,002,238	7,413	1,193,314	4,758	14,716,422
**Other Proceeds [€]**	1,241	203,406	1,611	252,965	1,426	228,186	426	1,318,951
**Total Revenue [€]**	9,684	1,588,096	7,995	1,255,203	8,840	1,421,650	5,184	16,035,373
**Cover [€]**	632	103,728	92	14,436	362	59,082	107	330,501
**Coverage [%]**	**7**		**1**		**4**		**2**	

### Crohn’s Disease–main and secondary diagnoses, procedures and DRGs

**Fig 1:**
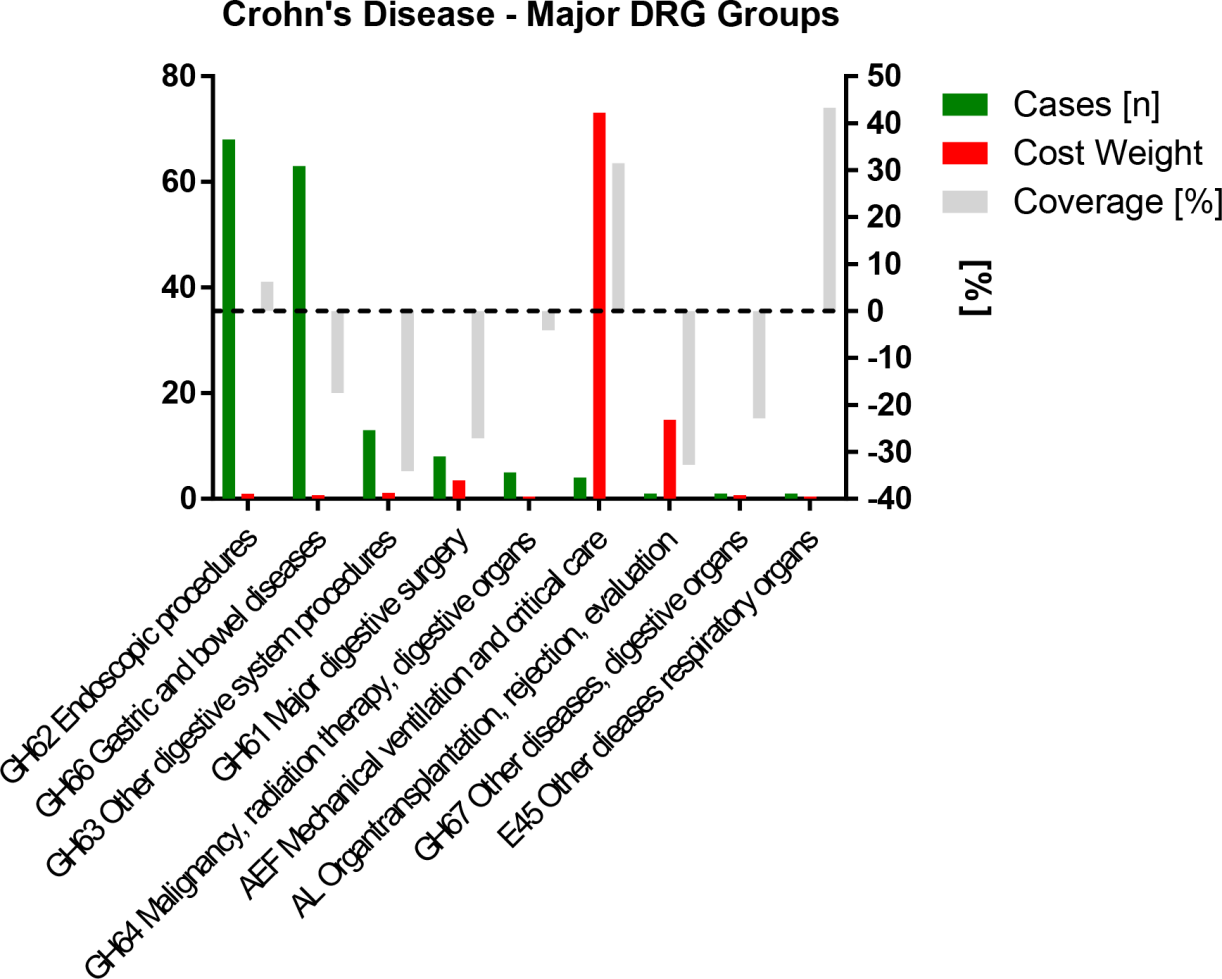
Crohn’s Disease: Major DRG categories sorted by case frequency and stratified by cost weight (CW) and coverage.

All 164 Crohn’s disease patients were grouped into 9 main DRG categories (**Fig 1**) and 33 actual DRGs (**S1 Table**).

This grouper assignment was based on 29 main diagnoses (**S2 Table**), 388 distinct secondary diagnoses (**S3 Table**) of and 167 distinct procedures (**S4 Table**).

In most patients (n = 129 78.66%) a Crohn’s disease ICD-10 code (K50.0 group) was selected as the main (i.e. discharge) diagnosis indicating admission for actual worsening of their illness. The list of secondary diagnoses shows that many patients were immunosuppressed, screened and treated for opportunistic infections or experienced other complications of their disease and treatment such as diarrhea induced hypokalemia, intestinal obstruction, fistulas, urinary tract infections, bleeding anemia, and required surgery, central line placement or isolation for contamination with multiresistant pathogens. The most commonly performed procedures were endoscopies with biopsies mostly in intravenous anesthesia, pelvic and abdominal MRIs or CTs with contrast, the administration of biologics and other immune therapies, surgical procedures including fistula seton placement, transfusions of various blood products as well as central line placement. The large number of almost 400 different secondary diagnoses along with 167 distinct procedures and interventions performed is consistent with the diverse nature and course of Crohn’s disease itself, its generally higher number of associated extraintestinal manifestations[24, 25] and the tertiary care type of setting in our center. The medical complexity of these cases is only partly reflected in the DRGs calculated by the grouper software, which defines cost weight largely by invasive procedures and critical care. The majority of cases fell into deficient DRGs. **Fig 1** Our analysis demonstrates that only cases that required an endoscopy or were admitted to the ICU were profitable, while those assigned to native gastroenterology DRGs were not. The excess coverage of critical care and particularly mechanical ventilation plus non-DRG proceeds eventually generated an overall positive mean cover statement of +7%. **Table 1**

### Crohn’s disease–comprehensive cost and revenue analysis

#### Actual costs

The analysis of the mean actual costs (9051€) (**S5 Table**) shows that on average most expenditures were accrued in the medical ward, intensive care unit and laboratory cost centers and spent for personnel (3982€), followed by materials (including medications) (2999€) and for infrastructure (2070€).

Within personnel expenses for nursing care of Crohn’s patients exceeded compensation for physicians, while in the materials section medications and in the infrastructure section non-medical infrastructure costs (administrative overhead, utilities, short-term maintenance, software licenses etc.) dominated.

#### DRG Proceeds

The mean actual costs of Crohn’s disease care (9051€) were not balanced by mean DRG proceeds (8441€) (**S6 Table**). There was a substantial mismatch between major cost centers and types. While the DRG overall personnel coverage of 4665€ exceeded the actual expenses, the budget for personnel expenses on the medical ward fell fifty percent short and exceeded the actual expenses on the intensive care unit. There was also a major gap in materials coverage (including medications) (1610€). Infrastructure costs (2176€) appeared overall balanced, but a closer look revealed a mismatch here too.

#### Non-DRG Proceeds

The detected mismatch, especially in the personnel budget of the ICU and medical ward and the non-medical infrastructure was not compensated with extra 34€ and 8€ respectively of non-DRG proceeds as these are mainly meant to cover novel medications, diagnostic tests and procedures (1200€). **S7 Table**

#### Total Coverage

**Fig 2:**
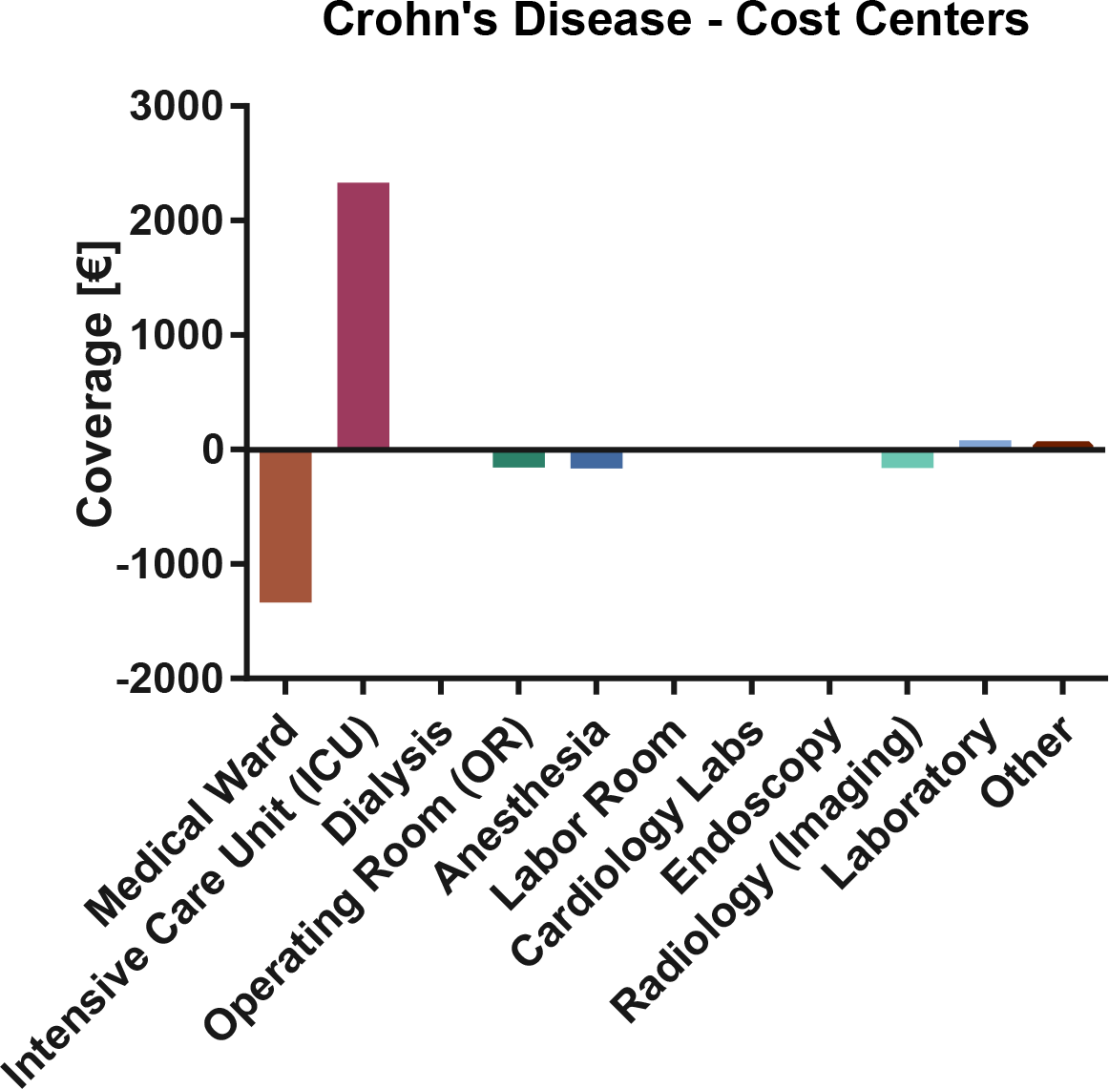
Crohn’s Disease: Cost center based total coverage analysis. Bars denote mean excess or deficit.

In a comparison of actual costs with DRG proceeds plus non-DRG proceeds, the most deficient cost centers remain the medical ward, operating room, imaging and anesthesia, while the ICU generated the largest excess coverage. **Fig 2**

**Fig 3:**
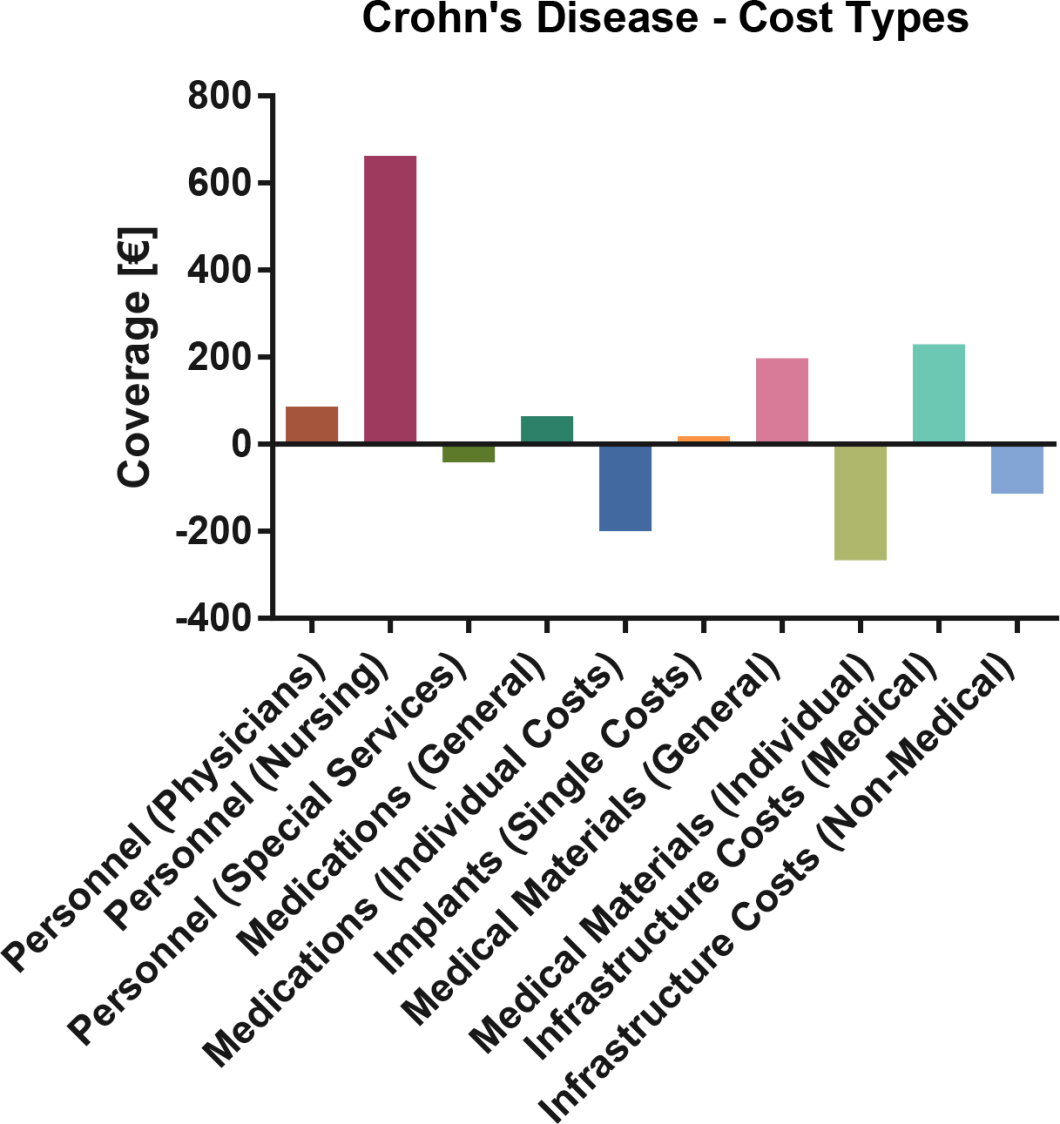
Crohn’s Disease: Cost type based total coverage analysis. Bars denote mean excess or deficit.

At cost type level, the largest excess coverage was observed in the (ICU) nursing budget, whereas individual medication and material costs and non-medical infrastructure costs contributed the most to the deficit. **Fig 3.**

### Ulcerative colitis–main and secondary diagnoses, procedures and DRGs

**Fig 4:**
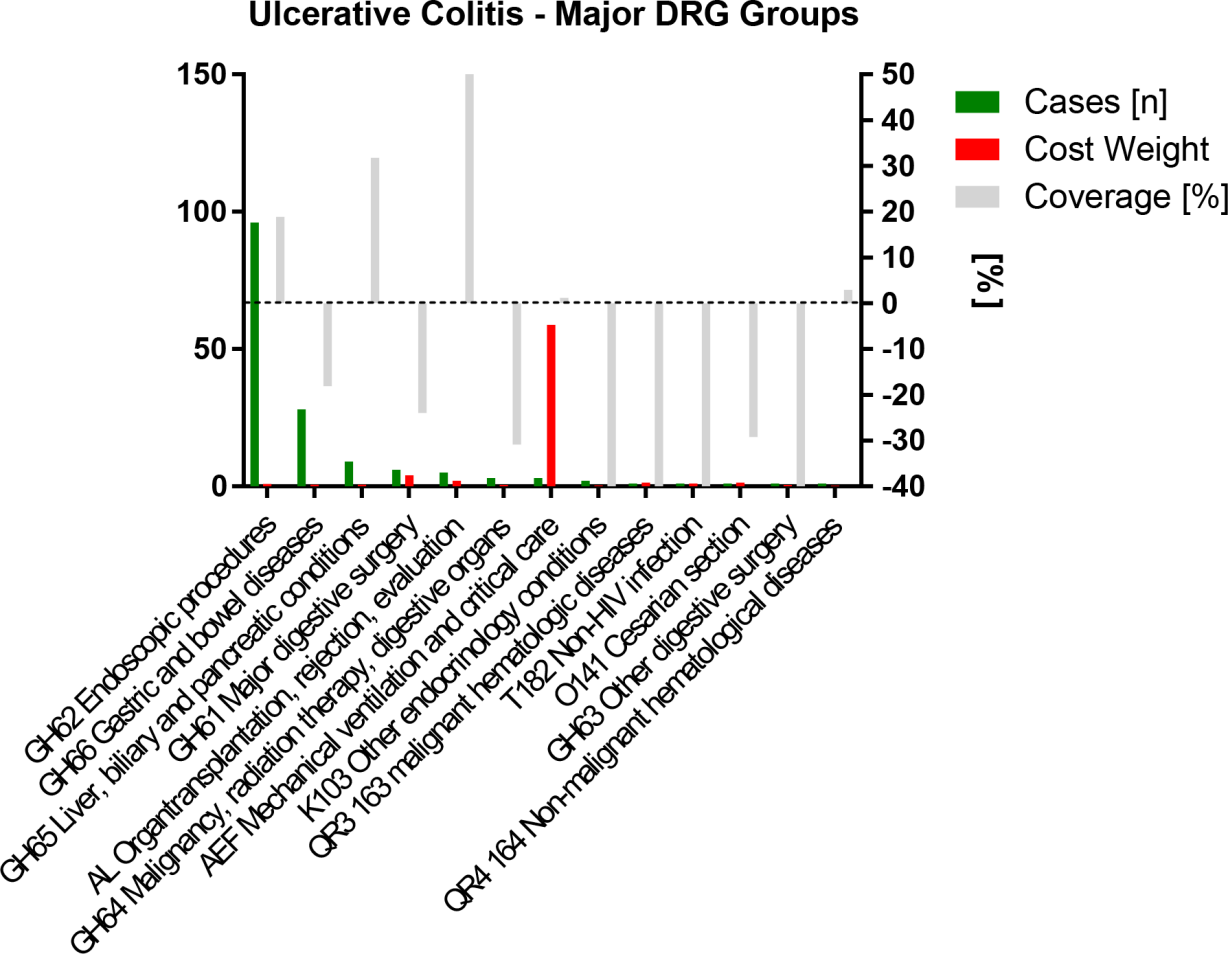
Ulcerative Colitis: Major DRG categories sorted by case frequency and stratified by cost weight (CW) and coverage.

All 157 ulcerative colitis patients were grouped into 13 main DRG categories (Fig 4) and 28 actual DRGs (S8 Table).

This grouper assignment was based on 31 main diagnoses (**S9 Table**), 295 distinct secondary diagnoses (**S10 Table**) and 225 distinct procedures (**S11 Table**).

In half of the patients (n = 77 49.04%) an ulcerative colitis ICD-10 code (K51 group) was selected as the main (i.e. discharge) diagnosis, in more than a third (n = 53 33.75%) a (primary sclerosing) cholangitis ICD-10 code (K83 K81 groups) and in 7.64% of cases (n = 12) another liver (including transplant) problem related ICD-10 code (K74, K76, T86 groups). This indicates that 98.49% of patients were admitted for either their UC or a UC related liver problem.

The list of secondary diagnoses shows that many patients were immunosuppressed and developed infections, came in for primary sclerosing cholangitis (PSC) related issues including evaluation for or status post liver transplantation, required screening and treatment for opportunistic infections or experienced other complications of their disease and treatment such as bleeding anemia, iron deficiency anemia, coagulation problems, surgery or isolation for contamination or infection with multiresistant pathogens.

The most commonly performed procedures were upper and lower endoscopies as well as hepatobiliary interventions in mostly intravenous anesthesia (including papillotomy, biliary balloon dilation, biliary stenting, biopsies and cytology, and variceal banding), abdominal, pelvic MRI and CT or MRCP with contrast, the administration of biologics and other immune therapies, transfusion of blood products as well as central line placement.

The large number of almost 300 different secondary diagnoses along with 225 distinct procedures and interventions performed is consistent with the tertiary care type of setting of our institution which is also the nation’s highest volume visceral transplant center.

**Fig 5:**
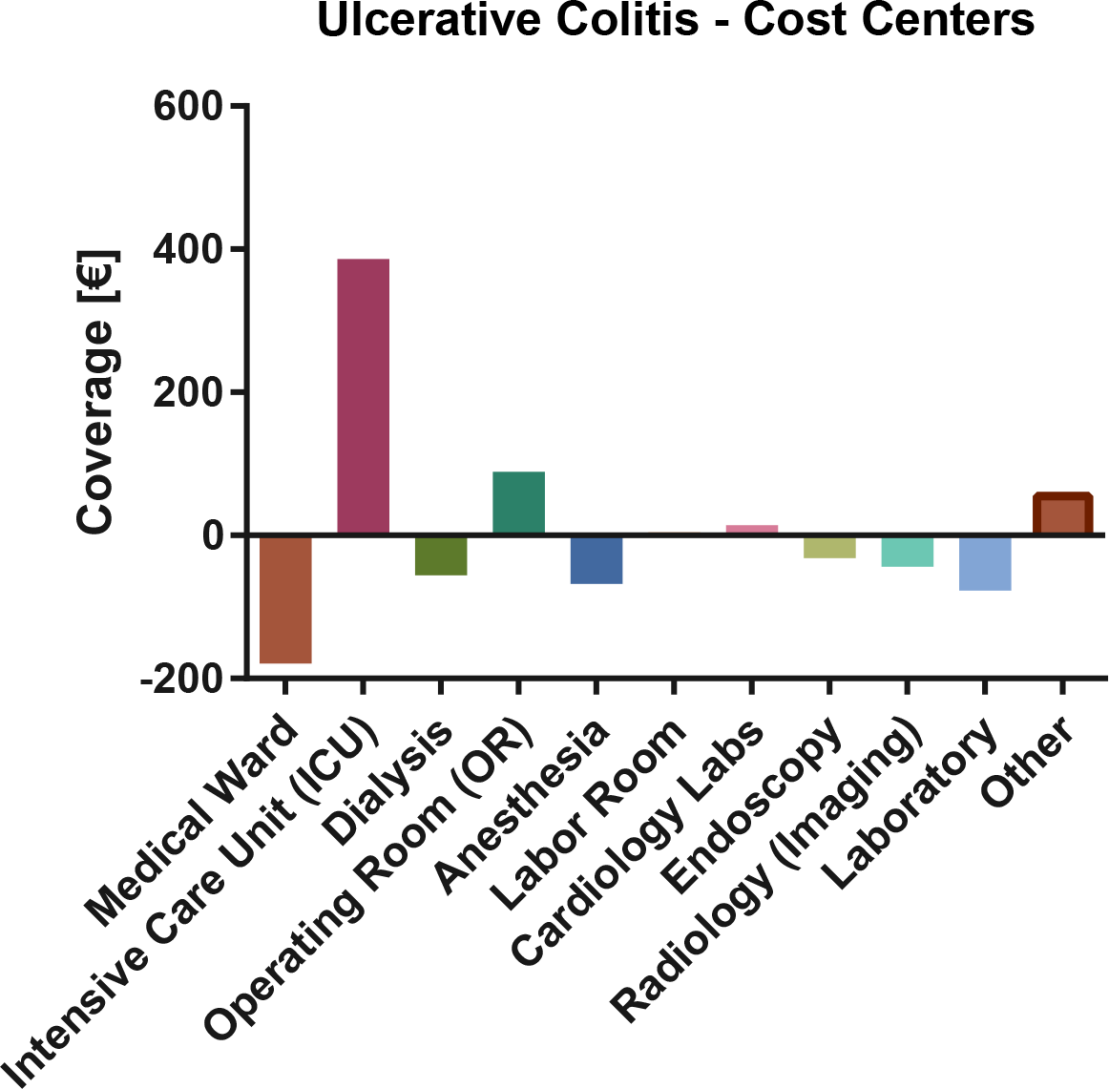
Ulcerative colitis: Cost center based total coverage analysis. Bars denote mean excess or deficit.

The medical complexity of these cases is better reflected in the DRGs calculated by the grouper software, which defines cost weight largely by invasive procedures such as ERCP and critical care than in Crohn’s disease. Most cases fell into fairly well covered DRGs. The DRG coverage distribution was more even than in Crohn’s disease with fewer extremes. **Fig 5** demonstrates that cases requiring an endoscopy or affected the hepatobiliary system were profitable, while those assigned to digestive surgery DRGs or DRGs with malignancy were highly deficient. The excess coverage of hepatobiliary and complex endoscopy cases plus non-DRG proceeds eventually generated an overall positive mean cover statement of +2%. **Table 1**

### Ulcerative colitis–comprehensive cost and revenue analysis

#### Actual costs

The analysis of the mean actual costs (7903€) (**S12 Table**) shows that on average most expenses were accrued in the medical ward, intensive care unit and laboratory cost centers and were spent for personnel (3192€), closely followed by materials (including medications) (3207€) and infrastructure costs (1502€).

Within personnel, the expenditures for nursing care of ulcerative colitis patients exceeded those for physicians, whereas in the materials section medications and in the infrastructure section non-medical infrastructure costs (administrative overhead) dominated. Of note, like in Crohn’s disease more money was spent for non-medical infrastructure costs than for physicians.

#### DRG-Proceeds

The mean actual costs of ulcerative colitis were not balanced by mean DRG proceeds (6384€) (**S13 Table**). The mismatch was largely caused by insufficient coverage of personnel costs on the medical ward and materials. This becomes even more obvious when comparing DRG proceeds (1307€) and actual costs for materials which do not recover individual medications and medical materials expenditures.

#### Non-DRG Proceeds

The detected mismatch, was partly compensated by non-DRG materials (1431€) proceeds as these are mainly meant to cover novel medications, diagnostic tests and procedures. (S14 Table).

#### Total Coverage

**Fig 6:**
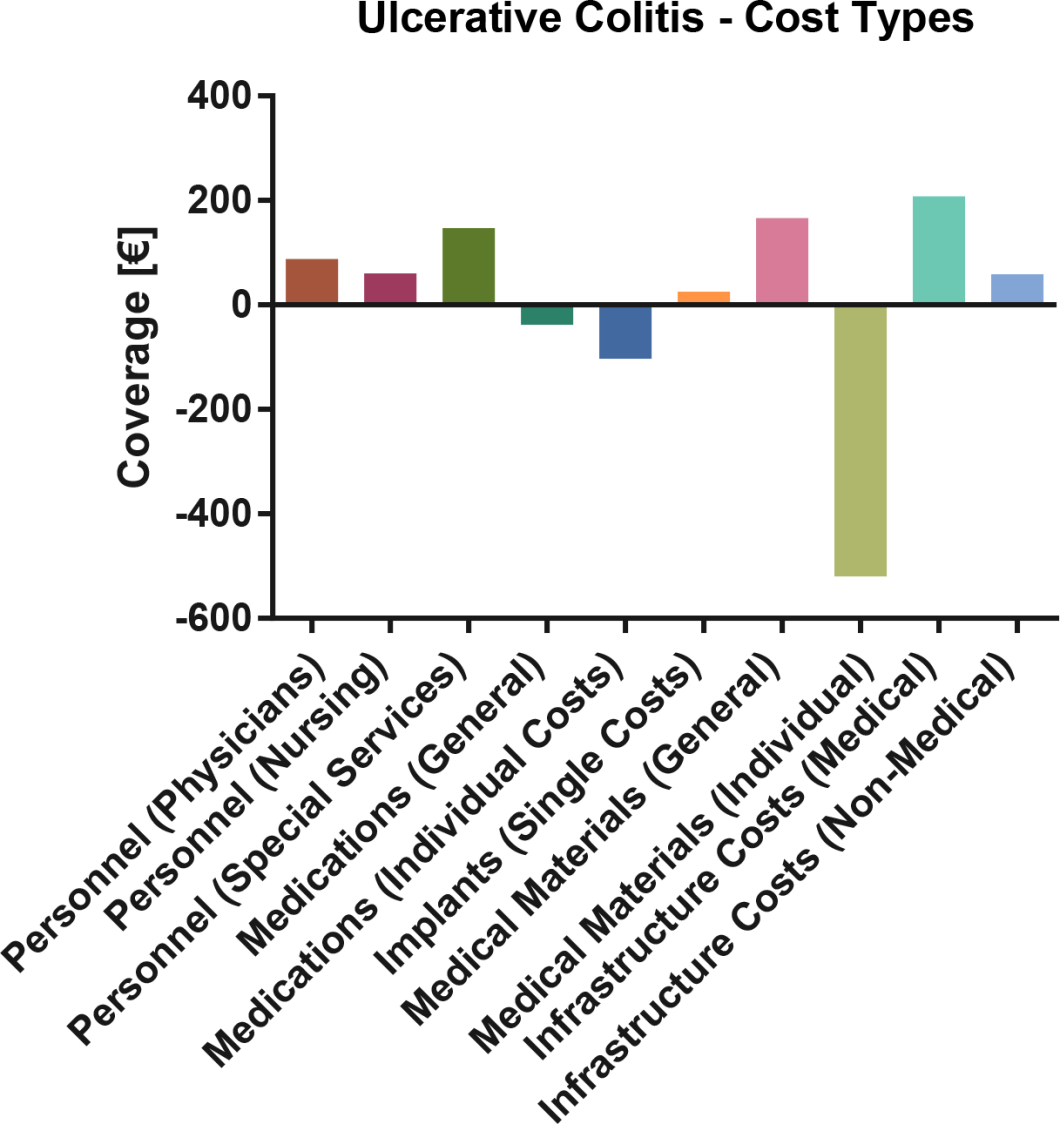
Ulcerative Colitis: Cost type based total coverage analysis. Bars denote mean excess or deficit.

In a comparison of actual costs with DRG plus non-DRG proceeds, the most deficient cost centers remain the medical ward, dialysis, anesthesia and endoscopy, while the ICU generated a modest excess coverage. **Fig 5** At cost type level, the largest excess coverage was generated through the medical infrastructure budget, whereas individual medication and medical material costs contributed the most to the deficit. **Fig 6**

### Relation of coverage to gender, patient complexity level (PCCL) and length of stay

**Fig 7:**
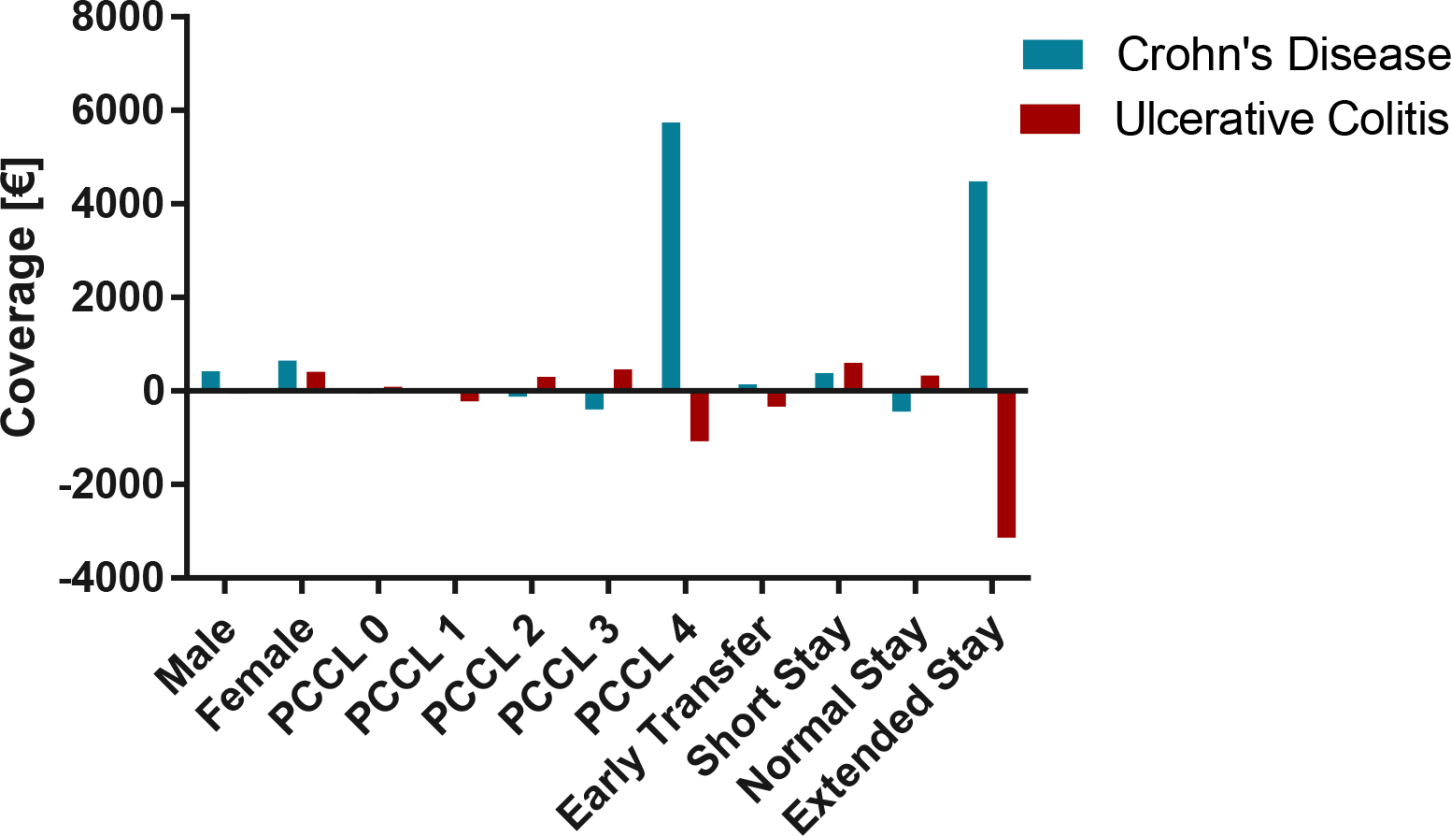
Relation of mean total coverage to gender, patient complexity level (PCCL) and length of stay stratified by Crohn’s disease and ulcerative colitis.

Relating coverage to length of stay, PCCL and gender revealed that very sick patients (PCCL 4), extended stays and female gender in CD as opposed to mild to moderately sick (PCCL 2 and PCCL 3) patients, short stays and female gender in UC were associated with excess coverage. **Fig 7**

### Individual medications and materials analysis at single cost level

Individual medication (CD vs. UC: 514 vs. 449 distinct medication formulations) and medical material (CD 324 vs. UC: 222 distinct medical materials) costs contribute second most to the expenditures of academic IBD inpatient care (**S5 Table and S12 Table**). The also make up most of the remaining deficit in total coverage (**Fig 1** and **Fig 2**). Thus, we looked at them in Crohn’s disease (detailed data on file) and ulcerative colitis (detailed data on file) in more detail.

#### Individual medical materials

In Crohn’s disease, the most expensive medical materials were used for interventional endoscopy and radiology, critical care and dialysis as well as medical imaging (CT and MRI contrast agents) (detailed data on file). In ulcerative colitis, the expenditure distribution was comparable with additional spending for artificial extracorporeal liver support.

#### Individual medications

In Crohn’s disease anti-TNF biologics, packed red blood cells and antifungals are the three most expensive medications, while in ulcerative colitis antifungals, platelets and anti-TNF biologics lead (detailed data on file). These lists once again illustrate the severity of the illness of academic IBD inpatients who are obviously admitted with severe and often opportunistic infections frequently requiring the use of reserve antimicrobials including potent anti-fungal drugs, transfusion of blood products, parenteral nutrition or enteral nutrition supplementation and biologics.

### Discussion

This is the first and currently only comprehensive and highly detailed analysis of IBD inpatient care costs and revenues in a European DRG based healthcare system.

We show that IBD inpatients are on average more severely ill than their fellow inpatients as indicated by admissions for mostly life threatening complications of their disease such as fulminant flares, infections with multiresistant and/or opportunistic pathogens, acute bleeding and coagulation disorders, longer stays, requirement of more intense nursing and critical care, dependence on expensive cross sectional imaging studies with contrast agents, interventional endoscopy, blood products, immune therapies including biologics and special medical materials. Thus, perhaps not surprisingly the expenditures for their care are almost double of those for the average academic gastroenterology inpatient. Moreover, our detailed analyses demonstrate that costs, revenues, and their mismatch for Crohn’s disease and ulcerative colitis patients are distinct in various cost type and cost center domains, reemphasizing that CD and UC are two different systemic inflammatory disease groups, with Crohn’s being the most heterogeneous one.

We demonstrate that expenditures are not fully recovered by DRG proceeds. In extensive coverage analyses we show, that particularly in Crohn’s disease, but also in ulcerative many DRGs assigned by the grouper software result in coverage deficits. This is particularly true, when IBD specific, rather than interventional procedure oriented DRGs are assigned. Cost center and cost type analyses discovered particularly serious mismatches in in the personnel, laboratory, single medication and medical materials budgets. Blood products, antimicrobial and biologic drugs as well as interventional procedure and critical care related material costs contribute substantially to the deficit. Somewhat surprisingly, more money is spent for non-medical infrastructure costs than for physicians. This does not seem to be disease specific though, but rather relates to the size of the institution and a disproportional quota of controlling and business vs. medical staff. Non-DRG generated proceeds were almost double that of the average gastroenterology and hepatology inpatient (16.1% vs. 8.2%) and contribute to an overall positive cover statement for both UC and CD. A positive cover statement should not be confused with a balanced cost and revenue distribution though, as our total coverage analyses conclude. PCCL and length of stay have literally the opposite effects on total coverage in CD and UC, while female gender appears to independently associated with greater revenues.

While several studies have theoretically modeled and estimated healthcare costs of IBD patients [3, 4] [26], very few have investigated *actual* DRG data. In 1990, new incentives regarding delivery of inpatient care by physicians and administrators have resulted from Medicare's DRG-based prospective reimbursement system in the US. A research study published that year investigated the hypothetical application of the IBD Medicare DRG on 507 IBD inpatient admissions (of which only 10.8% were actual Medicare cases) staying on average 9.71 days at a US large tertiary care hospital and concluded inadequate reimbursement with an average theoretical loss of 127.24 US$ which largely resulted from CD patients requiring surgery[27].

A Canadian study compared direct patient care costs between 1994 and 1995 based on Canada’s DRG system[9] for medical and surgical IBD admissions. Surgical admissions were more costly, especially when controlling for total parenteral nutrition. Interestingly, the non-digestive DRG coded admissions, were more costly, which is the opposite of what we found, probably because the G-DRG[23] grouper puts more weight on interventional procedures and ICU care. However, regarding cost centers and cost types, the Canadian data is strikingly in line with ours listing nursing, medications, labs, operating room, diagnostic imaging and endoscopy as the top categories[28].

In their 2005 and 2006 data based case control study the same group compared also IBD with non-IBD age and gender matched costs[29]. Our data can be partially related to the inpatient portion of their study that compared 1089 IBD cases with 5279 controls within one Canadian province. They reported a mean total cost (excluding administrative overheads, emergency room visits and physician salaries) for IBD 13495 CAN$, CD 12940 CAN$, UC 14183 CAN$ and non-IBD 12607 CAN$ respectively. Since their non-IBD inpatient population consisted of controls from an entire health system irrespective of diagnosis and specialty no conclusions can be drawn here. However, their report that medications and biologics again contributed largely to costs is in line with our results. These finding are apparently independent of any coding related issues as Canadian doctors are well aware of and properly use DRG coding in IBD[30].

Our study has limitations. Due to its single center design and restriction to an academic adult gastroenterology and hepatology department it may not fully reflect the situation in other academic medical centers or patients cared for in other subspecialties, i.e. pediatric IBD patients or IBD patients exclusively cared for in the surgery department or the situation in community hospitals, which don’t carry the extra effort and expenses of transplant patients. Although the German DRG system was derived and refined from the Australian DRG system, which was modeled after the US Medicare DRG system, national specifics in healthcare funding preclude the full extrapolation of numeric data and uncritical applicability of the results to all other countries, which applies to virtually all health economic analyses published to date. Owed to the data source and privacy legislation we were unable to phenotype patients according to the Montreal classification[31], report their disease onset or disease duration, individual medications, their medical and surgical history details or outpatient encounters (where no DRG billing is applied in Germany), which also impact on their overall all healthcare costs.

Moreover, DRGs and their reimbursement are not static. In fact, many countries annually review and revise their DRGs to accommodate the continuous advance of the science, practice and economics of medicine[32] [33]. Partial aspects of this process are reflected in our study. The non-DRG proceeds include adjustments to balance the higher medication costs not currently covered by DRGs. However, science and practice advance much faster than the administration and distribution of healthcare funds.

Future research needs to investigate the development of academic inpatient care costs and revenues over time, study academic outpatient care costs and compare both to private and community in- and outpatient care data. A multicenter setting at national level is highly desirable, but currently not feasible as access to reliable primary data is very complicated due to privacy legislation and political partisanship of different providers and payors.

DRGs were thought to improve efficiency because they provide incentives for hospitals to limit the services per patient and to treat more patients, but also in (un)intended consequences, such as reduced quality of care, preference for profitable cases (“cherry picking”), forced patient transfers (“patient dumping”), and frequent readmissions[33]. Our data demonstrate, that all native, non-procedure or critical care oriented IBD DRGs are still deficient, despite their annual adjustment and creation of almost 40% new DRGs since their introduction in 2004. Academic medical centers like ours rightfully receiving and caring for these complex diseases are taking an economic downturn, because even one of the most sophisticated and refined DRG systems is still unable to address their trues needs and associated costs. Patient “dumping” from other providers that refer patients to academic medical centers (who cannot reject patients by law) when their DRGs are financially exhausted is an everyday reality and applies to IBD. Patient organizations representing these and other rare conditions need to encourage politicians, payors and hospital management organizations to act on this to avoid an increasingly negative impact on the quality and availability of their well- deserved care.

## Supporting Information

S1 Table Crohn’s Disease–DRGs and key economic figures. (DOCX)

S2 Table Crohn’s disease–all coded main diagnoses. (DOCX)

S3 Table Crohn’s disease—top 25 coded secondary diagnoses (out of 388). (DOCX)

S4 Table Crohn’s disease–top 25 coded procedures at case and procedure level (out of 167). (DOCX)

S5 Table Crohn’s disease–costs analysis showing actual costs grouped by cost types and cost centers. (DOCX)

S6 Table Crohn’s disease–costs analysis showing mean DRG-proceeds grouped by cost types and cost centers. (DOCX)

S7 Table Crohn’s disease–costs analysis showing non-DRG proceeds grouped by cost types and cost centers. (DOCX)

S8 Table Ulcerative colitis–DRGs and key economic figures. (DOCX)

S9 Table Ulcerative colitis–all coded main diagnoses. (DOCX)

S10 Table Ulcerative Colitis–top 25 coded secondary diagnoses (out of 295). (DOCX)

S11 Table Ulcerative colitis–top 25 procedures (out of 225). (DOCX)

S12 Table Ulcerative colitis—costs analysis showing actual costs grouped by cost types and cost centers. (DOCX)

S13 Table Ulcerative colitis—costs analysis showing mean DRG-proceeds grouped by cost types and cost centers. (DOCX)

S14 Table Ulcerative colitis–costs analysis showing non-DRG proceeds grouped by cost types and cost centers. (DOCX)

## References

[1] Baumgart DC, Sandborn WJ. Crohn's disease. Lancet. 2012;380(9853):1590–605. 10.1016/S0140-6736(12)60026-9 . 22914295

[2] Danese S, Fiocchi C. Ulcerative colitis. N Engl J Med. 2011;365(18):1713–25. 10.1056/NEJMra1102942 . 22047562

[3] Cohen RD, Yu AP, Wu EQ, Xie J, Mulani PM, Chao J. Systematic review: the costs of ulcerative colitis in Western countries. AlimentPharmacolTher. 2010;31(7):693–707. 10.1111/j.1365-2036.2010.04234.x . 20064142

[4] Yu AP, Cabanilla LA, Wu EQ, Mulani PM, Chao J. The costs of Crohn's disease in the United States and other Western countries: a systematic review. CurrMedResOpin. 2008;24(2):319–28. 10.1185/03007990SX260790 . 18067689

[5] Busse R, World Health O, Regional Office for E, EuroDrg. Diagnosis-related groups in Europe moving towards transparency, efficiency and quality in hospitals Maidenhead: Open University Press; 2011. 10.1136/bmj.f3197 23747967

[6] Hospital Healthcare Europe: European Hospital and Healthcare Federation (HOPE); 2014 [6.1.2016]. Available: http://content.yudu.com/Library/A2tz92/HHE2014/resources/index.htm?referrerUrl=http%3A%2F%2Ffree.yudu.com%2Fitem%2Fdetails%2F1864882%2FHHE-2014.

[7] Schreyögg J, Stargardt T, Tiemann O, Busse R. Methods to determine reimbursement rates for diagnosis related groups (DRG): A comparison of nine European countries. Health Care Manag Sci. 2006;9(3):215–23. 10.1007/s10729-006-9040-1 17016927

[8] US Healthcare Cost and Utilization Project (HCUP): DRG: Agency for Healthcare Research Quality (AHQR); 2015 [6.1.2016]. Available: http://www.hcup-us.ahrq.gov/db/vars/siddistnote.jsp?var=drg.

[9] Sutherland JM. Hospital payment mechanisms: An overview and options for Canada: Canadian Health Services Research Foundation; 2015.

[10] Australian refined diagnosis-related groups (AR-DRG) data cubes (AIHW): Australian Institute of Health and Welfare; 2015 [6.1.2016]. Available: http://www.aihw.gov.au/hospitals-data/ar-drg-data-cubes/#ARDRGs.

[11] Fetter RB, Thompson JD, Mills RE. A system for cost and reimbursement control in hospitals. Yale J Biol Med. 1976;49(2):123–36. 941461 PMC2595277

[12] Quentin W, Scheller-Kreinsen D, Blumel M, Geissler A, Busse R. Hospital payment based on diagnosis-related groups differs in Europe and holds lessons for the United States. Health Aff (Millwood). 2013;32(4):713–23. 10.1377/hlthaff.2012.0876 . 23569051

[13] Union E. Community framework for state aid for research and development and innovation. Official Journal of the European Union. 2006;C323(1):1–26.

[14] Porter ME, Guth C. Redefining German Health Care: Defining the Problem. 1st ed. Berlin Heidelberg: Springer; 2015 5–24 p.

[15] Shiralkar AP, Bharat P, Shreekant. SAP NetWeaver BW 7.3—Practical Guide: SAP PRESS; 2012 2012-11-28. 789 p.

[16] The International Statistical Classification Of Diseases And Related Health Problems, 10th revision, German Modification (ICD-10-GM) [Text]. DIMDI—German Institute of Medical Documentation and Information; 2015 [6.1.2016]. Available: https://www.dimdi.de/static/en/klassi/icd-10-gm/index.htm.

[17] WHO. International Statistical Classification of Diseases and Related Health Problems 10th Revision 2015 [28.2.2015]. Available: http://apps.who.int/classifications/icd10/browse/2015/en.

[18] German modification (OPS) of the international classification of health interventions (ICHI): DIMDI—German Institute of Medical Documentation and Information; 2015 [23.12.2015]. Available: https://www.dimdi.de/static/de/klassi/ops/kodesuche/onlinefassungen/opshtml2015/index.htm.

[19] International Classification of Health Interventions (ICHI): WHO-FIC Family Development Committee; 2014 [6.1.2016]. Alpha 2 2014:[Available: http://sydney.edu.au/health-sciences/ncch/docs/Ichipkg2014.exe.

[20] Preyra C. Coding response to a case-mix measurement system based on multiple diagnoses. Health Serv Res. 2004;39(4 Pt 1):1027–45. 10.1111/j.1475-6773.2004.00270.x 15230940 PMC1361050

[21] Rogowski JR, Byrne DJ. Comparison of alternative weight recalibration methods for diagnosis-related groups. Health Care Financ Rev. 1990;12(2):87–101. 10113568 PMC4193111

[22] National Coding Guidelines: German Institute for the Hospital Remuneration System (InEK); 2015 [6.1.2016]. Available: http://www.g-drg.de/cms/content/view/full/5064.

[23] G-DRG: German Institute for the Hospital Remuneration System (InEK); 2015 [6.1.2015]. Available: http://www.g-drg.de/cms/content/download/5415/41977/version/3/file/Fallpauschalenkatalog_2015_140923.pdf?pk_campaign=Sys15&pk_kwd=FPKPdf.

[24] Vavricka SR, Brun L, Ballabeni P, Pittet V, Prinz Vavricka BM, Zeitz J, et al Frequency and risk factors for extraintestinal manifestations in the Swiss inflammatory bowel disease cohort. AmJGastroenterol. 2011;106(1):110–9. 10.1038/ajg.2010.343 . 20808297

[25] Ott C, Obermeier F, Thieler S, Kemptner D, Bauer A, Scholmerich J, et al The incidence of inflammatory bowel disease in a rural region of Southern Germany: a prospective population-based study. EurJGastroenterolHepatol. 2008;20(9):917–23. 10.1097/MEG.0b013e3282f97b33 . 18794607

[26] van der Valk ME, Mangen MJ, Leenders M, Dijkstra G, van Bodegraven AA, Fidder HH, et al Healthcare costs of inflammatory bowel disease have shifted from hospitalisation and surgery towards anti-TNFalpha therapy: results from the COIN study. Gut. 2014;63(1):72–9. 10.1136/gutjnl-2012-303376 . 23135759

[27] Vulgaropulos SP, Lyle CB, Sessions JT. Potential economic impact of applying DRG-based prospective payment categories to inflammatory bowel disease patients. Dig Dis Sci. 1990;35(5):577–81. . 2110054 10.1007/BF01540404

[28] Bernstein CN, Papineau N, Zajaczkowski J, Rawsthorne P, Okrusko G, Blanchard JF. Direct hospital costs for patients with inflammatory bowel disease in a Canadian tertiary care university hospital. Am J Gastroenterol. 2000;95(3):677–83. 10.1111/j.1572-0241.2000.01845.x . 10710056

[29] Bernstein CN, Longobardi T, Finlayson G, Blanchard JF. Direct medical cost of managing IBD patients: a Canadian population-based study. Inflamm Bowel Dis. 2012;18(8):1498–508. 10.1002/ibd.21878 . 22109958

[30] Farrokhyar F, McHugh K, Irvine EJ. Self-reported awareness and use of the International Classification of Diseases coding of inflammatory bowel disease services by Ontario physicians. Can J Gastroenterol. 2002;16(8):519–26. . 12226679 10.1155/2002/619574

[31] Silverberg MS, Satsangi J, Ahmad T, Arnott ID, Bernstein CN, Brant SR, et al Toward an integrated clinical, molecular and serological classification of inflammatory bowel disease: Report of a Working Party of the 2005 Montreal World Congress of Gastroenterology. Can J Gastroenterol. 2005;19 Suppl A:5–36. . 16151544 10.1155/2005/269076

[32] Scheller-Kreinsen D, Quentin W, Busse R. DRG-based hospital payment systems and technological innovation in 12 European countries. Value Health. 2011;14(8):1166–72. 10.1016/j.jval.2011.07.001 . 22152189

[33] Busse R, Geissler A, Aaviksoo A, Cots F, Hakkinen U, Kobel C, et al Diagnosis related groups in Europe: moving towards transparency, efficiency, and quality in hospitals? BMJ. 2013;346:f3197 10.1136/bmj.f3197 . 23747967

