# Supplementary material for: The Expenditures for Academic Inpatient Care of Inflammatory Bowel Disease Patients Are Almost Double Compared with Average Academic Gastroenterology and Hepatology Cases and Not Fully Recovered by Diagnosis-Related Group (DRG) Proceeds
Source: PLoS One. 2016 Jan 19;11(1):e0147364. doi: 10.1371/journal.pone.0147364 (PMC4718463; doi:10.1371/journal.pone.0147364)
Supplement: S1 Table — (DOCX) [file pone.0147364.s001.docx]

**S1 Table Crohn’s Disease – DRGs and key economic figures**

| **DRG** | **DRG Text** | **n** | **Cost**  **Weight**  **(CW)** | **Length**  **Of Stay**  **[days]** | **Total**  **Costs** | **Daily**  **Costs** | **DRG**  **Proceeds** | **Other**  **Proceeds** | **Mean**  **Coverage**  **including**  **Other Proceeds** | **Mean Coverage**  **including**  **Other**  **Proceeds**  **[%]** |
| --- | --- | --- | --- | --- | --- | --- | --- | --- | --- | --- |
| G48B | Colonoscopy with catastrophic or severe CC, with complicating procedure or age < 15 years, without severe gut infection, except status post organ transplantation | 51 | 0.98 | 5.7 | 2,965 € | 521 € | 2,34 € | 377 € | 347 € | 11.7 % |
| G64C | Inflammatory bowel disease , age > 17 and age < 70 years | 43 | 0.45 | 4.0 | 2,609 € | 652 € | 1,331 € | 944 € | -335 € | -12.8 % |
| G26Z | Other operations on anus | 9 | 0.79 | 10.0 | 5,816 € | 582 € | 2,372 € | 1,474 € | -1,970 € | -33.9 % |
| G64A | Inflammatory bowel disease or other serious diseases of the digestive organs , with extremely severe CC | 7 | 1.27 | 13.1 | 6,058 € | 461 € | 3,790 € | 884 € | -1,384 € | -22.8 % |
| G47Z | Other gastroscopy of major diseases of the digestive organs , without extremely severe or severe CC or gastroscopy without little complex gastroscopy, age <15 years | 6 | 0.68 | 2.7 | 2,117 € | 794 € | 2,040 € | 0 € | -78 € | -3.7 % |
| H41C | Complex treatment of multidrug-resistant pathogens in diseases and disorders of the digestive organs | 5 | 0.79 | 5.0 | 2,704 € | 541 € | 2,358 € | 44 € | -301 € | -11.1 % |
| G77Z | Complex therapeutic ERCP without extremely severe or severe CC , without photodynamic therapy , age > 2 years without complex intervention , or other ERCP | 5 | 1.93 | 17.6 | 8,387 € | 477 € | 5,757 € | 1,171 € | -1,460 € | -17.4 % |
| H41B | Malignant neoplasm of digestive organs, a day of occupancy or without extremely severe CC | 4 | 0.94 | 3.3 | 2,198 € | 676 € | 2,806 € | 331 € | 939 € | 42.7 % |
| G60B | Complex therapeutic ERCP with heavy CC without photodynamic therapy or age <3 years or more complex intervention | 4 | 0.42 | 2.0 | 2,063 € | 1,032 € | 1,254 € | 1,102 € | 292 € | 14.1 % |
| G64B | Work on small and large intestine except for congenital malformation or age > 1 year, without highly complex operation , without complicating diagnosis , with very complex intervention | 3 | 0.21 | 1.0 | 1,745 € | 1,745 € | 636 € | 829 € | -280 € | -16.1 % |
| G18B | Inflammatory bowel disease , age < 18 years or age > 69 years | 3 | 2.37 | 11.0 | 8,767 € | 797 € | 7,063 € | 0 € | -1,705 € | -19.4 % |
| A09B | Complex rectum or rectal resection with certain other intervention , regardless of specific liver metastases surgery, without complicating constellation | 2 | 41.47 | 93.5 | 127,719 € | 1,366 € | 123,780 € | 12,143 € | 8,204 € | 6.4 % |
| G12C | Esophagitis , gastroenteritis, gastrointestinal bleeding , ulcer disease and various diseases of the digestive organs with certain complicating diagnosis or complex procedure or with dialysis | 2 | 1.04 | 9.5 | 5,526 € | 582 € | 3,094 € | 0 € | -2,432 € | -44.0 % |
| G67A | Other OR procedures on the digestive organs without complex or overly complex OR procedure | 2 | 2.82 | 35.0 | 10,982 € | 314 € | 8,421 € | 0 € | -2,562 € | -23.3 % |
| G16B | Ventilation > 499 and <1000 hours of complex OR procedure or polytrauma or intensive medical complex treatment > 3430/3220 effort points , without highly complex procedure , age > 15 years , complicated with very complex intervention or constellation | 2 | 6.15 | 54.0 | 30,392 € | 563 € | 18,358 € | 0 € | -12,034 € | -39.6 % |
| A07C | Liver transplantation with mechanical ventilation > 59 and <180 hours or with graft rejection or with combined kidney | 1 | 127.31 | 299.0 | 297,539 € | 995 € | 380,005 € | 43,061 € | 125,527 € | 42.2 % |
| A09C | Various interventions on the hepatobiliary system with extremely severe CC or complex intervention | 1 | 82.15 | 193.0 | 171,441 € | 888 € | 245,218 € | 12,628 € | 86,404 € | 50.4 % |
| G18C | Colonoscopy with extremely severe or severe CC , complicating intervention or age <15 years , with severe intestinal infection or condition after organ transplantation | 1 | 2.31 | 10.0 | 6,318 € | 632 € | 6,907 € | 0 € | 590 € | 9.3 % |
| E69F | Esophagitis , gastroenteritis, gastrointestinal bleeding , ulcer disease and various diseases of the digestive organs with complex diagnostic or extremely severe CC or age <3 years or > 74 or severe CC at certain diagnosis | 1 | 0.45 | 3.0 | 929 € | 310 € | 1,331 € | 0 € | 402 € | 43.3 % |
| G65Z | Other OR procedures on the digestive organs with moderately complex OR procedure | 1 | 0.53 | 4.0 | 1,353 € | 338 € | 1,573 € | 0 € | 220 € | 16.3 % |
| G67C | Adhesiolysis in the peritoneum , age < 4 years with extremely severe or severe CC or small work on small and large intestine with extremely severe CC , age > 5 years without spec. PTAs of abdominal vessels | 1 | 0.27 | 2.0 | 652 € | 326 € | 800 € | 0 € | 148 € | 22.7 % |
| G46C | Malignant neoplasm of hepatobiliary system and pancreas, a day of occupancy or without complex diagnosis or without extremely severe CC | 1 | 0.96 | 4.0 | 4,175 € | 1,044 € | 2,872 € | 0 € | -1,304 € | -31.2 % |
| G71Z | Work on small and large intestine except for congenital malformation or age > 1-year highly complex intervention or complicating diagnosis | 1 | 0.70 | 11.0 | 5,936 € | 540 € | 2,092 € | 2,486 € | -1,357 € | -22.9 % |
| G18A | Other moderately severe diseases of the digestive organs | 1 | 3.24 | 14.0 | 13,635 € | 974 € | 9,668 € | 2,486 € | -1,480 € | -10.9 % |
| H61B | Various gastroscopy without little complex gastroscopies of major diseases of the digestive organs , with severe CC or non-fatal diseases of the digestive organs , with catastrophic, or severe CC in children , without esophageal perforation | 1 | 0.61 | 5.0 | 3,481 € | 696 € | 1,833 € | 0 € | -1,648 € | -47.3 % |
| G04B | Esophagitis , gastroenteritis, gastrointestinal bleeding , ulcer disease and various diseases of the digestive organs without complex procedure , without complex diagnosis, without extremely severe CC | 1 | 2.93 | 12.0 | 10,490 € | 874 € | 8,731 € | 0 € | -1,758 € | -16.8 % |
| G12B | Obstruction of the digestive tract | 1 | 1.90 | 26.0 | 12,951 € | 498 € | 5,674 € | 4,147 € | -3,130 € | -24.2 % |
| G67B | Bronchitis and bronchial asthma, age > 5 years, one of occupancy or age > 5 years and age < 56 years old, without extremely severe or severe CC or signs and respiratory symptoms without complex diagnosis, age > 15 years , or during hyperventilation | 1 | 0.57 | 5.0 | 5,192 € | 1,038 € | 1,695 € | 0 € | -3,496 € | -67.3 % |
| G48A | Work on small and large intestine except for congenital malformation or age > 1 year, without highly complex operation , without complicating diagnosis , without very complex engagement with complex intervention | 1 | 1.64 | 27.0 | 12,545 € | 465 € | 4,901 € | 2,155 € | -5,489 € | -43.8 % |
| H12A | Ventilation > 499 and < 1000 hrs. with complicated OR procedures or polytrauma or internal medicine complex treatment, > 3430/3220 points, without complicating constellation, Age > 15 years , or without complicated OR procedure, without polytrauma, with complicating constellation or complex medical treatment 2352/2209 - 3430/3220 points or age < 16 years | 1 | 4.10 | 50.0 | 19,069 € | 381 € | 12,230 € | 0 € | -6,840 € | -35.9 % |
| A01B | Ventilation > 999 and < 1,800 hours with complex OR procedure , without polytrauma , without complicating constellation , age > 15 years , or without complex OR procedure or polytrauma , age > 15 years , with critical care, complex treatment > 2352/2208 points | 1 | 14.96 | 49.0 | 94,530 € | 1,929 € | 44,653 € | 18,896 € | -30,981 € | -32.8 % |

*All data denote means.
