# Supplementary material for: The Expenditures for Academic Inpatient Care of Inflammatory Bowel Disease Patients Are Almost Double Compared with Average Academic Gastroenterology and Hepatology Cases and Not Fully Recovered by Diagnosis-Related Group (DRG) Proceeds
Source: PLoS One. 2016 Jan 19;11(1):e0147364. doi: 10.1371/journal.pone.0147364 (PMC4718463; doi:10.1371/journal.pone.0147364)
Supplement: S2 Table — (DOCX) [file pone.0147364.s002.docx]

**S2 Table Crohn’s disease – all coded main diagnoses**

| **ICD** | **Text** | **n** | **%** | **Coverage** |
| --- | --- | --- | --- | --- |
| **K50.0** | Crohn disease of small intestine | 42 | 25.6 % | **-117 €** |
| **K50.1** | Crohn disease of large intestine | 39 | 23.8 % | **-536 €** |
| **K50.82** | Crohn disease of the esophagus and the gastrointestinal tract affecting multiple parts | 30 | 18.3 % | **3,146 €** |
| **K50.88** | Other Crohn disease | 15 | 9.1 % | **-3,266 €** |
| **C18.1** | Malignant neoplasm of colon: Appendix | 4 | 2.4 % | **292 €** |
| **K83.1** | Obstruction of bile duct | 4 | 2.4 % | **127 €** |
| **K50.9** | Crohn disease, unspecified | 3 | 1.8 % | **958 €** |
| **K62.4** | Stenosis of anus and rectum | 2 | 1.2 % | **1,277 €** |
| **K83.0** | Cholangitis | 2 | 1.2 % | **238 €** |
| **K86.0** | Alcohol-induced chronic pancreatitis | 2 | 1.2 % | **1,264 €** |
| **K91.2** | Postsurgical malabsorption, not elsewhere classified | 2 | 1.2 % | **-4,577 €** |
| **K92.2** | Gastrointestinal hemorrhage, unspecified | 2 | 1.2 % | **-3,871 €** |
| **A09.0** | Other and unspecified gastroenteritis and colitis of infectious origin | 1 | 0.6 % | **534 €** |
| **A09.9** | Gastroenteritis and colitis of unspecified origin | 1 | 0.6 % | **774 €** |
| **B37.81** | Candida esophagitis | 1 | 0.6 % | **-3,762 €** |
| **C17.0** | Malignant neoplasm: Duodenum | 1 | 0.6 % | **-1,304 €** |
| **C25.4** | Malignant neoplasm: Endocrine pancreas | 1 | 0.6 % | **-1,648 €** |
| **D12.6** | Benign neoplasm: Colon, unspecified | 1 | 0.6 % | **1,662 €** |
| **J80** | Adult respiratory distress syndrome | 1 | 0.6 % | **125,527 €** |
| **K31.5** | Obstruction of duodenum | 1 | 0.6 % | **1,433 €** |
| **K31.88** | Other specified diseases of the stomach and the duodenum | 1 | 0.6 % | **1,051 €** |
| **K55.0** | Acute vascular disorders of intestine | 1 | 0.6 % | **-19,847 €** |
| **K56.6** | Other and unspecified intestinal obstruction | 1 | 0.6 % | **-3,404 €** |
| **K56.7** | Ileus, unspecified | 1 | 0.6 % | **220 €** |
| **K61.0** | Anal abscess | 1 | 0.6 % | **-1,357 €** |
| **K61.1** | Rectal abscess | 1 | 0.6 % | **-1,272 €** |
| **K80.31** | Gallstone with cholangitis and biliary obstruction | 1 | 0.6 % | **-1,262 €** |
| **K85.01** | Idiopathic acute pancreatitis with organ complications | 1 | 0.6 % | **-6,840 €** |
| **R06.0** | Dyspnea | 1 | 0.6 % | **402 €** |
