# Supplementary material for: The Expenditures for Academic Inpatient Care of Inflammatory Bowel Disease Patients Are Almost Double Compared with Average Academic Gastroenterology and Hepatology Cases and Not Fully Recovered by Diagnosis-Related Group (DRG) Proceeds
Source: PLoS One. 2016 Jan 19;11(1):e0147364. doi: 10.1371/journal.pone.0147364 (PMC4718463; doi:10.1371/journal.pone.0147364)
Supplement: S3 Table — (DOCX) [file pone.0147364.s003.docx]

**S3 Table Crohn’s disease - top 25 coded secondary diagnoses (out of 388)**

| **ICD** | **Text** | **n** | **%** |
| --- | --- | --- | --- |
| **Z11** | Special screening examination for infectious and parasitic diseases | 52 | 31.7 % |
| **D90** | Immune compromise due to radiation, chemotherapy or other immunosuppressive measures | 51 | 31.1 % |
| **I10.90** | Essential (primary) hypertension | 26 | 15.9 % |
| **K56.6** | Other and unspecified intestinal obstruction | 25 | 15.2 % |
| **K63.2** | Fistula of intestine | 24 | 14.6 % |
| **K50.88** | Other Crohn disease | 19 | 11.6 % |
| **E87.6** | Hypokalemia | 17 | 10.4 % |
| **Z29.0** | Isolation | 17 | 10.4 % |
| **N39.0** | Urinary tract infection, site not specified | 16 | 9.8 % |
| **Z43.2** | Attention to ileostomy | 16 | 9.8 % |
| **E53.8** | Deficiency of other specified B group vitamins | 14 | 8.5 % |
| **K21.0** | Gastro-esophageal reflux disease with esophagitis | 12 | 7.3 % |
| **Z43.3** | Attention to colostomy | 12 | 7.3 % |
| **Z45.20** | Adjustment and management of vascular access device | 12 | 7.3 % |
| **E03.8** | Other specified hypothyroidism | 11 | 6.7 % |
| **E11.90** | Non-insulin-dependent diabetes mellitus [Type-2-Diabetes]: no complications | 11 | 6.7 % |
| **K91.88** | Other digestive disease following medical measures, not specified elsewhere | 11 | 6.7 % |
| **K50.1** | Crohn disease of large intestine | 10 | 6.1 % |
| **T81.4** | Infection following a procedure, not elsewhere classified | 10 | 6.1 % |
| **Z90.4** | Acquired absence of other parts of digestive tract | 10 | 6.1 % |
| **D62** | Acute posthemorrhagic aaemia | 9 | 5.5 % |
| **Z22.3** | Carrier of other specified bacterial diseases | 9 | 5.5 % |
| **E73.8** | Other lactose intolerance | 8 | 4.9 % |
| **K50.0** | Crohn disease of small intestine | 8 | 4.9 % |
| **K57.30** | Diverticulosis of the colon without perforation, abscess or bleeding | 8 | 4.9 % |
