# Supplementary material for: The Expenditures for Academic Inpatient Care of Inflammatory Bowel Disease Patients Are Almost Double Compared with Average Academic Gastroenterology and Hepatology Cases and Not Fully Recovered by Diagnosis-Related Group (DRG) Proceeds
Source: PLoS One. 2016 Jan 19;11(1):e0147364. doi: 10.1371/journal.pone.0147364 (PMC4718463; doi:10.1371/journal.pone.0147364)
Supplement: S4 Table — (DOCX) [file pone.0147364.s004.docx]

**S4 Table Crohn’s disease – top 25 coded procedures at case and procedure level (out of 167)**

| **OPS** | **Text** | **n**  **Case**  **Level** | **n**  **Procedure**  **Level** | **%** |
| --- | --- | --- | --- | --- |
| **8-900** | Intravenous anesthesia | 90 | 126 | 54.9 % |
| **1-650** | Diagnostic colonoscopy | 76 | 84 | 46.3 % |
| **1-444** | Endoscopic biopsy lower digestive tract | 71 | 73 | 43.3 % |
| **3-82a** | Pelvic MRI with contrast | 62 | 67 | 37.8 % |
| **3-825** | Abdominal MRI with contrast | 59 | 64 | 36.0 % |
| **6-001** | Administration of medications list 1 | 43 | 44 | 26.2 % |
| **1-632** | Diagnostic EGD | 33 | 39 | 20.1 % |
| **8-547** | Other immune therapy | 30 | 30 | 18.3 % |
| **1-440** | Endoscopic biopsy upper digestive tract, biliary tree or pancreas | 26 | 27 | 15.9 % |
| **5-469** | Other surgeries on the gut | 18 | 20 | 11.0 % |
| **3-225** | Abdominal CT with contrast | 16 | 30 | 9.8 % |
| **8-987** | Complex therapy for colonization or infection with multiresistant microbes | 15 | 15 | 9.1 % |
| **3-226** | Pelvic CT with contrast | 13 | 26 | 7.9 % |
| **8-930** | Monitoring of ventilation, heart and circulation with pulmonary artery pressure central venous pressure | 13 | 14 | 7.9 % |
| **8-800** | Transfusion of whole blood, packed red blood cells or platelets | 12 | 16 | 7.3 % |
| **5-513** | Endoscopic surgery on the biliary tree | 10 | 18 | 6.1 % |
| **5-491** | Surgical treatment of anal fistulas | 8 | 9 | 4.9 % |
| **5-98c** | Use of a stapler | 8 | 8 | 4.9 % |
| **8-810** | Transfusion of fresh frozen plasma and genetically manufactured plasma proteins | 8 | 10 | 4.9 % |
| **3-990** | Computerized image analysis with 3D rendering | 7 | 8 | 4.3 % |
| **8-176** | Therapeutic lavage of the abdominal cavity with drainage tubes in places and temporary abdominal closure | 7 | 7 | 4.3 % |
| **8-831** | Placement and change of central venous catheters | 7 | 25 | 4.3 % |
| **5-454** | Resection of small bowel | 6 | 8 | 3.7 % |
| **6-002** | Administration of medications from list 2 | 6 | 7 | 3.7 % |
| **8-854** | Hemodialysis | 6 | 31 | 3.7 % |
