# Supplementary material for: The Expenditures for Academic Inpatient Care of Inflammatory Bowel Disease Patients Are Almost Double Compared with Average Academic Gastroenterology and Hepatology Cases and Not Fully Recovered by Diagnosis-Related Group (DRG) Proceeds
Source: PLoS One. 2016 Jan 19;11(1):e0147364. doi: 10.1371/journal.pone.0147364 (PMC4718463; doi:10.1371/journal.pone.0147364)
Supplement: S5 Table — (DOCX) [file pone.0147364.s005.docx]

### **S5 Table** **Crohn’s disease – costs analysis showing actual costs grouped by cost types and cost centers**

| **Cost Groups** | Personnel  (Physicians) | Personnel  (Nursing) | Personnel  (Special Services) | Medications  (General) | Medications  (Individual Costs) | Implants  (Single Costs) | Medical Materials  (General) | Medical Materials (Individual) | Infrastructure Costs  (Medical) | Infrastructure Costs  (Non-Medical) | **Total** |
| --- | --- | --- | --- | --- | --- | --- | --- | --- | --- | --- | --- |
| Medical Ward | 671 | 973 | 78 | 189 | 730 |  | 70 | 31 | 137 | 870 | **3,748** |
| Intensive Care Unit (ICU) | 347 | 722 | 12 | 50 | 556 |  | 174 | 40 | 96 | 388 | **2,385** |
| Dialysis Unit | 9 | 28 | 1 | 5 |  |  | 4 | 23 | 5 | 10 | **85** |
| Operating Room (OR) | 141 |  | 182 | 8 | 0 | 28 | 109 | 41 | 45 | 131 | **685** |
| Anesthesia | 158 |  | 97 | 13 | 7 |  | 31 |  | 12 | 74 | **392** |
| Delivery Room |  |  |  |  |  |  |  |  |  |  |  |
| Cardiology Labs | 0 |  | 1 | 0 |  |  | 0 | 1 | 0 | 0 | **3** |
| Endoscopy | 44 |  | 69 | 3 | 0 | 5 | 48 | 73 | 29 | 56 | **327** |
| Radiology (Imaging) | 109 |  | 147 | 3 | 1 | 6 | 15 | 62 | 36 | 91 | **469** |
| Laboratory | 16 |  | 38 |  | 296 |  | 21 | 314 | 3 | 30 | **718** |
| Other | 67 | 0 | 73 | 5 |  |  | 39 | 0 | 9 | 46 | **239** |
| **Total** | **1,562** | **1,723** | **697** | **276** | **1,590** | **38** | **509** | **586** | **372** | **1,698** | **9,051** |
