# Supplementary material for: The Expenditures for Academic Inpatient Care of Inflammatory Bowel Disease Patients Are Almost Double Compared with Average Academic Gastroenterology and Hepatology Cases and Not Fully Recovered by Diagnosis-Related Group (DRG) Proceeds
Source: PLoS One. 2016 Jan 19;11(1):e0147364. doi: 10.1371/journal.pone.0147364 (PMC4718463; doi:10.1371/journal.pone.0147364)
Supplement: S6 Table — (DOCX) [file pone.0147364.s006.docx]

### **S6 Table** **Crohn’s disease – costs analysis showing mean DRG-proceeds grouped by cost types and cost centers**

| **Cost Groups** | Personnel  (Physicians) | Personnel  (Nursing) | Personnel  (Special Services) | Medications  (General) | Medications  (Individual Costs) | Implants  (Single Costs) | Medical Materials  (General) | Medical Materials (Individual) | Infrastructure Costs  (Medical) | Infrastructure Costs  (Non-Medical) | **Total** |
| --- | --- | --- | --- | --- | --- | --- | --- | --- | --- | --- | --- |
| Medical Ward | 325 | 566 | 28 | 68 | 27 | 0 | 52 | 10 | 156 | 533 | **1,765** |
| Intensive Care Unit (ICU) | 848 | 1798 | 36 | 247 | 128 | 0 | 368 | 45 | 273 | 691 | **4,435** |
| Dialysis Unit |  |  |  |  |  |  |  |  |  |  |  |
| Operating Room (OR) | 130 | 0 | 102 | 5 | 5 | 26 | 66 | 54 | 55 | 82 | **526** |
| Anesthesia | 94 | 0 | 59 | 7 | 2 | 0 | 22 | 0 | 13 | 27 | **226** |
| Delivery Room | 0 | 0 | 0 | 0 | 0 | 0 | 0 | 0 | 0 | 0 | **0** |
| Cardiology Labs | 2 | 0 | 0 | 0 | 0 | 6 | 1 | 4 | 1 | 2 | **19** |
| Endoscopy | 78 | 0 | 78 | 3 | 0 | 2 | 35 | 21 | 37 | 58 | **315** |
| Radiology (Imaging) | 60 | 0 | 65 | 1 | 1 | 14 | 14 | 44 | 30 | 49 | **277** |
| Laboratory | 40 | 0 | 157 | 5 | 103 | 0 | 130 | 73 | 21 | 78 | **606** |
| Other | 60 | 4 | 122 | 2 | 0 | 0 | 11 | 4 | 14 | 56 | **273** |
|  |  |  |  |  |  |  |  |  |  |  |  |
| **Total** | **1,637** | **2,367** | **651** | **339** | **267** | **49** | **700** | **256** | **599** | **1,577** | **8,441** |
