# Supplementary material for: The Expenditures for Academic Inpatient Care of Inflammatory Bowel Disease Patients Are Almost Double Compared with Average Academic Gastroenterology and Hepatology Cases and Not Fully Recovered by Diagnosis-Related Group (DRG) Proceeds
Source: PLoS One. 2016 Jan 19;11(1):e0147364. doi: 10.1371/journal.pone.0147364 (PMC4718463; doi:10.1371/journal.pone.0147364)
Supplement: S7 Table — (DOCX) [file pone.0147364.s007.docx]

### **S7 Table Crohn’s disease – costs analysis showing non-DRG proceeds grouped by cost types and cost centers**

| **Cost Groups** | Personnel  (Physicians) | Personnel  (Nursing) | Personnel  (Special Services) | Medications  (General) | Medications  (Individual Costs) | Implants  (Single Costs) | Medical Materials  (General) | Medical Materials (Individual) | Infrastructure Costs  (Medical) | Infrastructure Costs  (Non-Medical) | **Total** |
| --- | --- | --- | --- | --- | --- | --- | --- | --- | --- | --- | --- |
| Medical Ward | 0 | 0 | 0 | 0 | 648 | 0 | 0 | 0 | 0 | 0 | **648** |
| Intensive Care Unit (ICU) | 0 | 1 | 0 | 0 | 262 | 0 | 0 | 18 | 0 | 0 | **281** |
| Dialysis Unit | 10 | 17 | 5 | 2 | 20 | 0 | 5 | 13 | 2 | 6 | **80** |
| Operating Room (OR) | 0 | 0 | 0 | 0 | 0 | 0 | 0 | 0 | 0 | 0 | **1** |
| Anesthesia | 0 | 0 | 0 | 0 | 0 | 0 | 0 | 0 | 0 | 0 | **1** |
| Delivery Room |  |  |  |  |  |  |  |  |  |  |  |
| Cardiology Labs |  |  |  |  |  |  |  |  |  |  |  |
| Endoscopy | 0 | 0 | 0 | 0 | 0 | 7 | 0 | 0 | 0 | 0 | **7** |
| Radiology (Imaging) | 0 | 0 | 0 | 0 | 0 | 0 | 0 | 30 | 0 | 0 | **30** |
| Laboratory | 0 | 0 | 0 | 0 | 194 | 0 | 1 | 1 | 0 | 0 | **195** |
| Other |  |  |  |  |  |  |  |  |  | 0 |  |
| **Total** | **11** | **18** | **5** | **2** | **1,123** | **7** | **5** | **63** | **2** | **6** | **1,242** |
