# Supplementary material for: The Expenditures for Academic Inpatient Care of Inflammatory Bowel Disease Patients Are Almost Double Compared with Average Academic Gastroenterology and Hepatology Cases and Not Fully Recovered by Diagnosis-Related Group (DRG) Proceeds
Source: PLoS One. 2016 Jan 19;11(1):e0147364. doi: 10.1371/journal.pone.0147364 (PMC4718463; doi:10.1371/journal.pone.0147364)
Supplement: S8 Table — (DOCX) [file pone.0147364.s008.docx]

**S8 Table Ulcerative colitis – DRGs and key economic figures***

| **DRG** | **DRG Text** | **n** | **Cost**  **Weight**  **(CW)** | **Length**  **Of Stay**  **[days]** | **Total**  **Costs** | **Daily**  **Costs** | **DRG**  **Proceeds** | **Other**  **Proceeds** | **Coverage**  **including**  **Other Proceeds** | **Coverage**  **including**  **Other**  **Proceeds**  **[%]** |
| --- | --- | --- | --- | --- | --- | --- | --- | --- | --- | --- |
| G48B | Colonoscopy with catastrophic or severe CC , complicating intervention or age <15 years , with complicating diagnosis without severe intestinal infection , except in state after organ transplantation | 40 | 1.03 | 7.7 | 3,430 € | 444 € | 3,077 € | 424 € | 71 € | 2.1 % |
| H41C | Complex therapeutic ERCP without extremely severe or severe CC , without photodynamic therapy , age > 2 years without complex intervention , or other ERCP | 33 | 0.69 | 2.4 | 1,493 € | 623 € | 2,067 € | 0 € | 574 € | 38.5 % |
| G64C | Inflammatory bowel disease , age > 17 and age < 70 years | 22 | 0.47 | 3.2 | 2,051 € | 644 € | 1,392 € | 741 € | 82 € | 4.0 % |
| H41B | Complex therapeutic ERCP with heavy CC without photodynamic therapy or age <3 years or more complex intervention | 8 | 1.16 | 4.3 | 2,098 € | 494 € | 3,471 € | 0 € | 1,373 € | 65.5 % |
| H41A | Complex therapeutic ERCP with extremely severe CC or photodynamic therapy | 8 | 1.47 | 5.5 | 2,973 € | 541 € | 4,383 € | 0 € | 1,411 € | 47.4 % |
| G16B | Complex rectum or rectal resection with certain other intervention , regardless of specific liver metastases surgery, without complicating constellation | 6 | 4.09 | 20.2 | 16,048 € | 796 € | 12,201 € | 0 € | -3,847 € | -24.0 % |
| G64A | Inflammatory bowel disease or other serious diseases of the digestive organs , with extremely severe CC | 6 | 1.67 | 21.7 | 8,138 € | 376 € | 4,996 € | 0 € | -3,142 € | -38.6 % |
| G48A | Colonoscopy with extremely severe or severe CC, complicating intervention or age <15 years , with severe intestinal infection or condition after organ transplantation | 4 | 0.86 | 3.0 | 1,556 € | 519 € | 2,557 € | 0 € | 1,001 € | 64.3 % |
| H60Z | Liver cirrhosis and certain non-infectious hepatitis with extremely severe CC | 4 | 0.80 | 3.0 | 1,237 € | 412 € | 2,375 € | 0 € | 1,139 € | 92.1 % |
| G47Z | Other gastroscopy of major diseases of the digestive organs , without extremely severe or severe CC or gastroscopy without little complex gastroscopy, age <15 years | 3 | 0.68 | 5.3 | 2,854 € | 535 € | 2,040 € | 0 € | -815 € | -28.5 % |
| G60B | Malignant neoplasm of digestive organs, a day of occupancy or without extremely severe CC | 3 | 0.53 | 5.3 | 2,296 € | 430 € | 1,586 € | 0 € | -710 € | -30.9 % |
| K64D | Endocrinopathies , age > 5 years, without complex diagnosis, without extremely severe CC | 2 | 0.45 | 5.0 | 3,676 € | 735 € | 1,342 € | 0 € | -2,334 € | -63.5 % |
| H64Z | Diseases of the gallbladder and bile ducts | 2 | 0.56 | 3.5 | 1,879 € | 537 € | 1,664 € | 0 € | -214 € | -11.4 % |
| A69Z | Evaluation stay prior to organ transplantation without recording on a waiting list | 2 | 2.38 | 10.5 | 4,797 € | 457 € | 7,092 € | 409 € | 2,705 € | 56.4 % |
| A07B | Ventilation > 999 and < 1,800 hours with complex OR procedure with polytrauma or complicating constellation or age <16 years , or without complex OR procedure , without polytrauma , age <16 years | 1 | 52.43 | 115.0 | 213,760 € | 1,859 € | 156,513 € | 48,815 € | -8,433 € | -3.9 % |
| A13E | Age > 1 year, with certain OR procedures or complicating constellation or critical care complex treatment 589/553 to 1176/1104 points or age < 16 years | 1 | 7.73 | 41.0 | 36,079 € | 880 € | 23,083 € | 5,081 € | -7,915 € | -21.9 % |
| R61H | Lymphoma and non- acute leukemia without sepsis, without complicating constellation , without agranulocytosis, without port implantation , without extremely severe CC , without complex diagnosis without bone affections , without complex diagnosis of leukemia | 1 | 1.39 | 18.0 | 9,341 € | 519 € | 4,146 € | 0 € | -5,195 € | -55.6 % |
| T60E | Sepsis without complicating constellation , except for state after organ transplantation , without complex diagnosis, without extremely severe CC , age > 9 years | 1 | 1.12 | 17.0 | 6,170 € | 363 € | 3,331 € | 0 € | -2,839 € | -46.0 % |
| O01E | Secondary cesarean section with complicating diagnosis, gestational age more than 33 completed weeks (SSW ) , without complex diagnosis | 1 | 1.38 | 13.0 | 5,803 € | 446 € | 4,107 € | 0 € | -1,696 € | -29.2 % |
| G26Z | Other operations on anus | 1 | 0.56 | 6.0 | 3,042 € | 507 € | 1,663 € | 0 € | -1,379 € | -45.3 % |
| H63C | Hepatic disorders except for malignant neoplasm , liver cirrhosis and certain non-infectious hepatitis , a day of occupancy or without complex diagnosis and without extremely severe or severe CC | 1 | 0.24 | 1.0 | 931 € | 931 € | 728 € | 0 € | -202 € | -21.7 % |
| Q61E | Erythrocyte disorders without complex diagnosis without aplastic anemia, without extremely severe CC |  | 0.27 | 1.0 | 786 € | 786 € | 809 € | 0 € | 23 € | 2.9 % |
| H63B | Hepatic disorders except for malignant neoplasm , liver cirrhosis and certain non-infectious hepatitis , more than one day , with complex diagnosis or extremely severe or severe CC , age > 0 years | 1 | 1.03 | 5.0 | 2,957 € | 591 € | 3,081 € | 0 € | 124 € | 4.2 % |
| H62B | Diseases of the pancreas except malignant neoplasm with acute pancreatitis, age > 15 years or liver cirrhosis and certain non-infectious hepatitis without extremely severe CC | 1 | 0.62 | 2.0 | 1,429 € | 714 € | 1,842 € | 0 € | 413 € | 28.9 % |
| A64Z | Evaluation stay prior to liver or kidney - pancreas transplant | 1 | 2.21 | 11.0 | 5,317 € | 483 € | 6,606 € | 0 € | 1,288 € | 24.2 % |
| A60C | Failure and rejection of an organ transplant , more than one day , without removal of an organ transplant , without complex OR procedure , without extremely severe CC , age > 15 years | 1 | 1.17 | 4.0 | 1,679 € | 420 € | 3,498 € | 0 € | 1,819 € | 108.3 % |
| A60A | Failure and rejection of an organ transplant , more than one day , with removal of an organ transplant or complex OR procedure or extremely severe CC | 1 | 2.40 | 6.0 | 2,190 € | 365 € | 7,158 € | 0 € | 4,968 € | 226.9 % |
| A18Z | Ventilation > 999 hours and transplantation of liver, lung, heart and bone marrow or stem cell transfusion | 1 | 116.24 | 191.0 | 486,697 € | 2,548 € | 346,961 € | 164,978 € | 25,242 € | - 1. % |

*All data denote means.
