# Supplementary material for: The Expenditures for Academic Inpatient Care of Inflammatory Bowel Disease Patients Are Almost Double Compared with Average Academic Gastroenterology and Hepatology Cases and Not Fully Recovered by Diagnosis-Related Group (DRG) Proceeds
Source: PLoS One. 2016 Jan 19;11(1):e0147364. doi: 10.1371/journal.pone.0147364 (PMC4718463; doi:10.1371/journal.pone.0147364)
Supplement: S9 Table — (DOCX) [file pone.0147364.s009.docx]

**S9 Table Ulcerative colitis – all coded main diagnoses**

| **ICD** | **Text** | **n** | **%** | **Coverage** |
| --- | --- | --- | --- | --- |
| **K51.8** | Other ulcerative colitis | 39 | 24.8 % | -452 € |
| **K83.1** | Obstruction of bile duct | 30 | 19.1 % | 1,060 € |
| **K83.0** | Cholangitis | 22 | 14.0 % | 1,733 € |
| **K51.0** | Ulcerative (chronic) pancolitis | 21 | 13.4 % | -1,347 € |
| **K51.2** | Ulcerative (chronic) proctitis | 7 | 4.5 % | 1,165 € |
| **K51.5** | Left sided colitis | 4 | 2.5 % | 299 € |
| **K51.3** | Ulcerative (chronic) rectosigmoiditis | 3 | 1.9 % | -76 € |
| **K51.9** | Ulcerative colitis, unspecified | 3 | 1.9 % | 336 € |
| **C18.1** | Malignant neoplasm: Appendix | 2 | 1.3 % | -902 € |
| **K72.0** | Acute and subacute hepatic failure | 2 | 1.3 % | -4,154 € |
| **K74.4** | Secondary biliary cirrhosis | 2 | 1.3 % | 1,033 € |
| **K74.6** | Other and unspecified cirrhosis of liver | 2 | 1.3 % | 1,245 € |
| **T86.41** | Chronic liver failure following transplantation | 2 | 1.3 % | 3,394 € |
| **A41.1** | Sepsis due to other specified staphylococcus | 1 | 0.6 % | -2,839 € |
| **C20** | Malignant neoplasm of rectum | 1 | 0.6 % | -325 € |
| **C82.1** | Follicular lymphoma grade II | 1 | 0.6 % | -5,195 € |
| **D12.3** | Benign neoplasm: Transverse colon | 1 | 0.6 % | 349 € |
| **D64.8** | Other specified anemias | 1 | 0.6 % | 23 € |
| **E16.1** | Other hypoglycemia | 1 | 0.6 % | -4,815 € |
| **E23.0** | Hypopituitarism | 1 | 0.6 % | 146 € |
| **K50.0** | Crohn disease of small intestine | 1 | 0.6 % | -398 € |
| **K63.5** | Polyp of colon | 1 | 0.6 % | 644 € |
| **K74.3** | Primary biliary cirrhosis | 1 | 0.6 % | 1,101 € |
| **K74.5** | Biliary cirrhosis, unspecified | 1 | 0.6 % | 413 € |
| **K75.0** | Abscess of liver | 1 | 0.6 % | -7,915 € |
| **K76.8** | Other specified diseases of liver | 1 | 0.6 % | -202 € |
| **K80.31** | Gallstone with cholangitis: with biliary obstruction | 1 | 0.6 % | 1,222 € |
| **K86.8** | Other specified diseases of pancreas | 1 | 0.6 % | 468 € |
| **K91.1** | Postgastric surgery syndromes | 1 | 0.6 % | -891 € |
| **K91.88** | Other digestive disease following medical measures, not specified elsewhere | 1 | 0.6 % | -1,003 € |
| **O68.0** | Labor and delivery complicated by fetal heart rate anomaly | 1 | 0.6 % | -1,696 € |
