# Supplementary material for: The Expenditures for Academic Inpatient Care of Inflammatory Bowel Disease Patients Are Almost Double Compared with Average Academic Gastroenterology and Hepatology Cases and Not Fully Recovered by Diagnosis-Related Group (DRG) Proceeds
Source: PLoS One. 2016 Jan 19;11(1):e0147364. doi: 10.1371/journal.pone.0147364 (PMC4718463; doi:10.1371/journal.pone.0147364)
Supplement: S10 Table — (DOCX) [file pone.0147364.s010.docx]

**S10 Table** **Ulcerative Colitis – top 25 coded secondary diagnoses (out of 295)**

| **ICD** | **Text** | **n** | **%** |
| --- | --- | --- | --- |
| **K51.8** | Other ulcerative colitis | 74 | 47.1 % |
| **D90** | Immune compromise due to radiation, chemotherapy or other immunosuppressive measures | 46 | 29.3 % |
| **K83.0** | Cholangitis | 44 | 28.0 % |
| **Z11** | Special screening examination for infectious and parasitic diseases | 42 | 26.8 % |
| **I10.90** | Essential hypertension: no hypertensive crisis | 24 | 15.3 % |
| **E87.6** | Hypokalemia | 22 | 14.0 % |
| **K83.1** | Obstruction of bile duct | 22 | 14.0 % |
| **K91.88** | Other digestive disease following medical measures, not specified elsewhere | 12 | 7.6 % |
| **K29.6** | Other gastritis | 11 | 7.0 % |
| **Z43.2** | Attention to ileostomy | 10 | 6.4 % |
| **D50.8** | Other iron deficiency anemias | 9 | 5.7 % |
| **E11.90** | Non-insulin-dependent diabetes mellitus [Type-2-Diabetes]: no complications | 9 | 5.7 % |
| **K74.6** | Other and unspecified cirrhosis of liver | 9 | 5.7 % |
| **Z90.4** | Acquired absence of other parts of digestive tract | 9 | 5.7 % |
| **Z94.4** | Liver transplant status | 9 | 5.7 % |
| **D62** | Acute posthaemorrhagic anaemia | 7 | 4.5 % |
| **K86.8** | Other specified diseases of pancreas | 7 | 4.5 % |
| **Z29.0** | Isolation | 7 | 4.5 % |
| **E03.8** | Other specified hypothyroidism | 6 | 3.8 % |
| **E78.5** | Hyperlipidemia, unspecified | 6 | 3.8 % |
| **I25.13** | Coronary artery disease: three vessels | 6 | 3.8 % |
| **D68.4** | Acquired coagulation factor deficiency | 5 | 3.2 % |
| **E86** | Volume depletion | 5 | 3.2 % |
| **T81.4** | Infection following a procedure, not elsewhere classified | 5 | 3.2 % |
| **Z88.1** | Personal history of allergy to other antibiotic agents | 5 | 3.2 % |
