# Supplementary material for: The Expenditures for Academic Inpatient Care of Inflammatory Bowel Disease Patients Are Almost Double Compared with Average Academic Gastroenterology and Hepatology Cases and Not Fully Recovered by Diagnosis-Related Group (DRG) Proceeds
Source: PLoS One. 2016 Jan 19;11(1):e0147364. doi: 10.1371/journal.pone.0147364 (PMC4718463; doi:10.1371/journal.pone.0147364)
Supplement: S11 Table — (DOCX) [file pone.0147364.s011.docx]

**S11 Table** **Ulcerative colitis – top 25 procedures (out of 225)**

| **OPS** | **Text** | **n**  **Case**  **Level** | **n**  **Procedure**  **Level** | **%** |
| --- | --- | --- | --- | --- |
| **8-900** | Intravenous anesthesia | 113 | 155 | 72.0 % |
| **1-444.7** | Endoscopic biopsies on the lower digestive tract: 1-5 biopsies | 42 | 42 | 26.8 % |
| **1-650.2** | Diagnostic colonoscopy. with ileoscopy | 33 | 33 | 21.0 % |
| **1-632** | Diagnostic EGD | 32 | 36 | 20.4 % |
| **1-640** | Diagnostic ERCP | 28 | 28 | 17.8 % |
| **3-825** | Abdominal MRI with contrast | 26 | 26 | 16.6 % |
| **1-650.0** | Partial diagnostic colonoscopy | 24 | 24 | 15.3 % |
| **5-513.d** | Endoscopic biliary procedures: dilation | 23 | 23 | 14.6 % |
| **1-444.6** | Endoscopic biopsies lower digestive tract: sequential biopsies | 21 | 21 | 13.4 % |
| **8-547.31** | Other immune therapy: immunosuppression: other mode of administration | 20 | 21 | 12.7 % |
| **1-440.a** | Endoscopic biopsies upper digestive tract. biliary tree and pancreas: 1 - 5 biopsies | 14 | 15 | 8.9 % |
| **3-225** | Abdominal CT with contrast | 14 | 39 | 8.9 % |
| **1-440.6** | Endoscopic biopsy upper digestive tract. biliary tree and pancreas | 13 | 13 | 8.3 % |
| **3-226** | Pelvic CT with contrast | 13 | 36 | 8.3 % |
| **3-843** | MRCP] | 12 | 12 | 7.6 % |
| **5-526.a** | Endoscopic pancreatic procedures: dilation | 12 | 12 | 7.6 % |
| **8-800.c0** | Transfusion of whole blood. packed red blood cells and platelets: 1 to 6 units | 12 | 12 | 7.6 % |
| **3-82a** | Pelvic MRI with contrast | 11 | 11 | 7.0 % |
| **5-429.a** | Other esophageal endoscopic procedures: banding of varices | 11 | 11 | 7.0 % |
| **5-513.f0** | Endoscopic biliary procedures: stenting | 10 | 11 | 6.4 % |
| **8-547.30** | Other immune therapy: immunosuppression: intravenous | 10 | 10 | 6.4 % |
| **6-001.e4** | Administration of medications from list 1 | 9 | 9 | 5.7 % |
| **5-513.1** | Endoscopic biliary procedures: papillotomy | 8 | 9 | 5.1 % |
| **5-513.a** | Endoscopic biliary procedures: dilation | 7 | 7 | 4.5 % |
| **8-831.0** | Placement and change of central venous catheters | 7 | 11 | 4.5 % |
