# Supplementary material for: The Expenditures for Academic Inpatient Care of Inflammatory Bowel Disease Patients Are Almost Double Compared with Average Academic Gastroenterology and Hepatology Cases and Not Fully Recovered by Diagnosis-Related Group (DRG) Proceeds
Source: PLoS One. 2016 Jan 19;11(1):e0147364. doi: 10.1371/journal.pone.0147364 (PMC4718463; doi:10.1371/journal.pone.0147364)
Supplement: S12 Table — (DOCX) [file pone.0147364.s012.docx]

### **S12 Table Ulcerative colitis - costs analysis showing actual costs grouped by cost types and cost centers**

| **Cost Groups** | Personnel  (Physicians) | Personnel  (Nursing) | Personnel  (Special Services) | Medications  (General) | Medications  (Individual Costs) | Implants  (Single Costs) | Medical Materials  (General) | Medical Materials (Individual) | Infrastructure Costs  (Medical) | Infrastructure Costs  (Non-Medical) | **Total** |
| --- | --- | --- | --- | --- | --- | --- | --- | --- | --- | --- | --- |
| Medical Ward | 440 | 583 | 45 | 114 | 291 |  | 39 | 9 | 99 | 553 | **2,172** |
| Intensive Care Unit (ICU) | 334 | 830 | 8 | 119 | 959 |  | 126 | 77 | 67 | 326 | **2,844** |
| Dialysis Unit | 31 | 90 | 4 | 14 |  |  | 12 | 161 | 15 | 31 | **356** |
| Operating Room (OR) | 93 |  | 101 | 4 | 0 | 12 | 56 | 39 | 25 | 68 | **399** |
| Anesthesia | 102 |  | 62 | 8 | 3 |  | 18 |  | 7 | 46 | **245** |
| Delivery Room | 1 |  | 2 | 0 |  |  | 0 | 0 | 0 | 1 | **4** |
| Cardiology Labs |  |  |  |  |  |  |  |  |  |  |  |
| Endoscopy | 55 |  | 89 | 4 |  | 2 | 62 | 135 | 39 | 70 | **456** |
| Radiology (Imaging) | 64 |  | 87 | 2 | 1 | 2 | 9 | 35 | 22 | 55 | **276** |
| Laboratory | 17 |  | 38 | 0 | 433 |  | 21 | 409 | 4 | 30 | **953** |
| Other | 68 | 0 | 52 | 5 |  |  | 24 | 3 | 7 | 37 | **196** |
| **Total** | **1,205** | **1,502** | **486** | **269** | **1,686** | **16** | **368** | **868** | **285** | **1,217** | **7,903** |
