# Supplementary material for: The Expenditures for Academic Inpatient Care of Inflammatory Bowel Disease Patients Are Almost Double Compared with Average Academic Gastroenterology and Hepatology Cases and Not Fully Recovered by Diagnosis-Related Group (DRG) Proceeds
Source: PLoS One. 2016 Jan 19;11(1):e0147364. doi: 10.1371/journal.pone.0147364 (PMC4718463; doi:10.1371/journal.pone.0147364)
Supplement: S13 Table — (DOCX) [file pone.0147364.s013.docx]

### **S13 Table** **Ulcerative colitis - costs analysis showing mean DRG-proceeds grouped by cost types and cost centers**

| **Cost Groups** | Personnel  (Physicians) | Personnel  (Nursing) | Personnel  (Special Services) | Medications  (General) | Medications  (Individual Costs) | Implants  (Single Costs) | Medical Materials  (General) | Medical Materials (Individual) | Infrastructure Costs  (Medical) | Infrastructure Costs  (Non-Medical) | **Total** |
| --- | --- | --- | --- | --- | --- | --- | --- | --- | --- | --- | --- |
| Medical Ward | 321 | 544 | 27 | 72 | 37 | 0 | 51 | 8 | 158 | 532 | **1,749** |
| Intensive Care Unit (ICU) | 481 | 953 | 35 | 134 | 151 | 5 | 208 | 20 | 165 | 387 | **2,539** |
| Dialysis Unit |  |  |  |  |  |  |  |  |  |  |  |
| Operating Room (OR) | 103 | 0 | 79 | 4 | 3 | 22 | 46 | 43 | 43 | 67 | **410** |
| Anesthesia | 72 | 0 | 42 | 7 | 3 | 0 | 17 | 1 | 11 | 22 | **175** |
| Delivery Room | 1 | 0 | 3 | 0 | 0 | 0 | 0 | 0 | 1 | 2 | **8** |
| Cardiology Labs | 2 | 0 | 2 | 0 | 0 | 1 | 1 | 4 | 1 | 2 | **14** |
| Endoscopy | 95 | 0 | 96 | 4 | 0 | 8 | 42 | 58 | 46 | 74 | **424** |
| Radiology (Imaging) | 50 | 0 | 58 | 1 | 1 | 3 | 12 | 36 | 27 | 44 | **232** |
| Laboratory | 40 | 0 | 154 | 3 | 94 | 2 | 126 | 64 | 21 | 78 | **582** |
| Other | 69 | 3 | 100 | 2 | 0 | 0 | 9 | 4 | 14 | 50 | **251** |
| **Total** | **1,235** | **1,500** | **597** | **226** | **290** | **41** | **512** | **238** | **488** | **1,257** | **6,384** |
