# Supplementary material for: The Expenditures for Academic Inpatient Care of Inflammatory Bowel Disease Patients Are Almost Double Compared with Average Academic Gastroenterology and Hepatology Cases and Not Fully Recovered by Diagnosis-Related Group (DRG) Proceeds
Source: PLoS One. 2016 Jan 19;11(1):e0147364. doi: 10.1371/journal.pone.0147364 (PMC4718463; doi:10.1371/journal.pone.0147364)
Supplement: S14 Table — (DOCX) [file pone.0147364.s014.docx]

**Supporting Information**

### **S14 Table** **Ulcerative colitis – costs analysis showing non-DRG proceeds grouped by cost types and cost centers**

| **Cost Groups** | Personnel  (Physicians) | Personnel  (Nursing) | Personnel  (Special Services) | Medications  (General) | Medications  (Individual Costs) | Implants  (Single Costs) | Medical Materials  (General) | Medical Materials (Individual) | Infrastructure Costs  (Medical) | Infrastructure Costs  (Non-Medical) | **Total** |
| --- | --- | --- | --- | --- | --- | --- | --- | --- | --- | --- | --- |
| Medical Ward | 0 | 0 | 0 | 0 | 244 | 0 | 0 | 0 | 0 | 0 | **244** |
| Intensive Care Unit (ICU) | 12 | 15 | 1 | 0 | 663 | 0 | 0 | 0 | 0 | 0 | **691** |
| Dialysis Unit | 34 | 47 | 28 | 5 | 71 | 0 | 23 | 70 | 4 | 19 | **300** |
| Operating Room (OR) | 9 | 0 | 7 | 0 | 22 | 0 | 0 | 40 | 0 | 0 | **79** |
| Anesthesia | 3 | 0 | 0 | 0 | 0 | 0 | 0 | 0 | 0 | 0 | **3** |
| Delivery Room |  |  |  |  |  |  |  |  |  |  |  |
| Cardiology Labs |  |  |  |  |  |  |  |  |  |  |  |
| Endoscopy |  |  |  |  |  |  |  |  |  |  |  |
| Radiology (Imaging) |  |  |  |  |  |  |  |  |  |  |  |
| Laboratory | 0 | 0 | 0 |  | 293 | 0 | 0 | 1 | 0 | 0 | **294** |
| Other |  |  |  |  |  |  |  |  |  |  |  |
| **Total** | **58** | **62** | **36** | **5** | **1,293** | **0** | **23** | **111** | **4** | **19** | **1,611** |
